# Supplementary material for: Assessing the relevance and uptake of core outcome sets (an agreed minimum collection of outcomes to measure in research studies) in Cochrane systematic reviews: a review
Source: BMJ Open. 2020 Sep 6;10(9):e036562. doi: 10.1136/bmjopen-2019-036562 (PMC7476465; doi:10.1136/bmjopen-2019-036562)
Supplement: Author's manuscript [file bmjopen-2019-036562.draft_revisions.pdf]

BMJ Open is committed to open peer review. As part of this commitment we make the peer review history of every article we publish publicly available.

When an article is published we post the peer reviewers' comments and the authors' responses online. We also post the versions of the paper that were used during peer review. These are the versions that the peer review comments apply to.

The versions of the paper that follow are the versions that were submitted during the peer review process. They are not the versions of record or the final published versions. They should not be cited or distributed as the published version of this manuscript.

BMJ Open is an open access journal and the full, final, typeset and author-corrected version of record of the manuscript is available on our site with no access controls, subscription charges or pay-per-view fees (http://bmjopen.bmj.com).

If you have any questions on BMJ Open's open peer review process please email info.bmjopen@bmj.com

BMJ Open

Assessing the relevance and uptake of core outcome sets in Cochrane systematic reviews: a review

Journal:	*BMJ Open*
Manuscript ID	bmjopen-2019-036562
Article Type:	Original research
Date Submitted by the Author:	19-Dec-2019
Complete List of Authors:	Williamson, Paula; University of Liverpool,<br>de Ávila Oliveira, Ricardo; Universidade Federal de Uberlândia<br>Clarke, Mike; Queen's University Belfast, Northern Ireland Methodology Hub<br>Gorst, Sarah L.; University of Liverpool<br>Hughes, Karen; University of Liverpool<br>Kirkham, Jamie J.; University of Liverpool, Biostatistics<br>Li, Tianjing; University of Colorado<br>Saldanha, Ian; Brown University<br>Schmitt, Jochen; Technische Universität Dresden, Department of Dermatology
Keywords:	STATISTICS & RESEARCH METHODS, Clinical trials < THERAPEUTICS, GENERAL MEDICINE (see Internal Medicine)

SCHOLARONE™
Manuscripts

I, the Submitting Author has the right to grant and does grant on behalf of all authors of the Work (as defined in the below author licence), an exclusive licence and/or a non-exclusive licence for contributions from authors who are: i) UK Crown employees; ii) where BMJ has agreed a CC-BY licence shall apply, and/or iii) in accordance with the terms applicable for US Federal Government officers or employees acting as part of their official duties; on a worldwide, perpetual, irrevocable, royalty-free basis to BMJ Publishing Group Ltd ("BMJ") its licensees and where the relevant Journal is co-owned by BMJ to the co-owners of the Journal, to publish the Work in this journal and any other BMJ products and to exploit all rights, as set out in our licence.

The Submitting Author accepts and understands that any supply made under these terms is made by BMJ to the Submitting Author unless you are acting as an employee on behalf of your employer or a postgraduate student of an affiliated institution which is paying any applicable article publishing charge ("APC") for Open Access articles. Where the Submitting Author wishes to make the Work available on an Open Access basis (and intends to pay the relevant APC), the terms of reuse of such Open Access shall be governed by a Creative Commons licence – details of these licences and which Creative Commons licence will apply to this Work are set out in our licence referred to above.

Other than as permitted in any relevant BMJ Author's Self Archiving Policies, I confirm this Work has not been accepted for publication elsewhere, is not being considered for publication elsewhere and does not duplicate material already published. I confirm all authors consent to publication of this Work and authorise the granting of this licence.

For peer review only

Assessing the relevance and uptake of core outcome sets in Cochrane systematic reviews: a review

Paula R Williamson[a*]

Ricardo de Ávila Oliveira[b]

Mike Clarke[3]

Sarah L Gorst[a]

Karen Hughes[a]

Jamie J Kirkham[d]

Tianjing Li[e]

Ian J Saldanha[f]

Jochen Schmitt[g]

[a] MRC North West Hub for Trials Methodology Research, University of Liverpool and member of Liverpool Health Partners, Liverpool, United Kingdom

[b] DECIR Faculdade de Medicina, Universidade Federal de Uberlândia, Uberlândia, Brazil

[c] Northern Ireland Methodology Hub, Centre for Public Health, Queen's University Belfast, Belfast, United Kingdom

[d] Centre for Biostatistics, Manchester Academic Health Science Centre, University of Manchester, Manchester, United Kingdom

[e] Department of Ophthalmology, University of Colorado Denver, Aurora, Colorado, USA

[f] Center for Evidence Synthesis in Health, Brown University School of Public Health, Providence, Rhode Island, USA

For peer review only

[g] Center for Evidence-based Healthcare, Medizinische Fakultät, Technische Univesität Dresden, Dresden, Germany

*Corresponding Author:**

Professor Paula Williamson

Department of Biostatistics

University of Liverpool

Block F Waterhouse Building, 1-5 Brownlow Street, Liverpool, L69 3GL, United Kingdom

Email: prw@liv.ac.uk

Tel: +44 (0) 151 794 9758

Word Count: 2625

For peer review only

For peer review only - http://bmjopen.bmj.com/site/about/guidelines.xhtml

ABSTRACT

Objectives: To examine the use of, and reference to the need for core outcome sets (COS) in Cochrane systematic reviews, and obtain the views of Cochrane Review Group Co-ordinating Editors on this topic.

Methods: A cohort of recent 2019 Cochrane systematic reviews were assessed for reference to the use of, or need for, a COS. Results were compared with existing COS published two years or more before the review publication. We asked 52 Cochrane Review Group Co-ordinating Editors about their involvement in COS development, CRG outcome selection policies, and their understanding of the advantages and challenges surrounding the standardisation of outcomes within their CRG, for example within Summary of Findings (SoF) tables.

Results: In the cohort of reviews, 40% (40/100) of reviewers noted problems due to outcome inconsistency across the included studies. In 7% (7/100) of reviews, a COS was referenced in relation to choice of outcomes for the review. Relevant COS could be considered for its update in 35% (33/93). Most responding editors (31/36, 86%) thought that COS should definitely or possibly be used in SoF tables.

Conclusions: Systematic reviewers are continuing to note outcome heterogeneity but are starting to use COS to inform their reviews. There is potential for greater uptake of COS in Cochrane reviews.

For peer review only - http://bmjopen.bmj.com/site/about/guidelines.xhtml

Keywords: Cochrane Review Group; Co-ordinating Editor; core outcome set; summary of findings; systematic reviews

STRENGHTS AND LIMITATIONS OF THIS STUDY

- A cohort of 100 Cochrane systematic reviews published in 2019 were assessed for use of COS
- Results for use of COS were compared with COS published two or more years earlier
- Views of Cochrane Editors were sought on the relevance of COS to systematic reviews

For peer review only

For peer review only - http://bmjopen.bmj.com/site/about/guidelines.xhtml

BACKGROUND

Systematic reviews of the effects of healthcare interventions summarise the available evidence and are used for decision making. Inconsistencies in the outcomes that are measured and reported, and the selective reporting of outcomes, in the included studies affect the robustness of the conclusions of a systematic review.[1 2] In addition, there is increasing awareness that differences exist between the outcomes measured in trials and outcomes that patients consider important. For example, only 50% of the outcomes that patients said matter to them were captured in the primary studies reviewed by the Canadian Agency for Drugs and Technologies in Health (CADTH) Common Drug Review.[3] Such problems contribute to research waste.[4]

One solution is for trialists in a particular topic area to measure and report a core outcome set (COS), which would then be used in reviews addressing that topic area. A COS is an agreed standardised collection of outcomes that should be measured and reported in a specific area of health. An additional dimension of the problem of outcome inconsistency relates to clinical trialists and systematic reviewers sometimes being interested in different types of outcomes.[5 6] Clarke and Williamson have argued for greater involvement of systematic reviewers in the development and implementation of COS.[1] They suggest this might help with planning the review as well as the selection of the specific outcomes to include in the Summary of Findings (SoF) tables that provide users of the review with the key quantitative findings. In a 2012 survey of Cochrane Review Group (CRG) Co-ordinating Editors (hereafter "editors", who oversee the production and maintenance of systematic reviews in particular areas of healthcare), the majority (33/45, 73%) thought that COS

For peer review only - http://bmjopen.bmj.com/site/about/guidelines.xhtml

should routinely be used in the SoF tables.[7] At that time, a third of the editors (14/45, 31%) reported personal involvement in the development of a COS.

The awareness and development of COS have substantially increased over recent years, with more than 100 new COS published since 2012 and at least 200 more whose development are ongoing.[8-13] A series of guidelines have been published to improve the design and reporting of COS studies.[14-17] The relevance of COS to defining review questions and planning the review, as well as the inclusion of patient-centred outcomes within COS, is acknowledged in the new Cochrane Handbook, which states, “where available, established sets of core outcomes should be used”.[18] One notable link between COS development and Cochrane is the work of the Cochrane Skin Group on the Cochrane Skin Core Outcome Set Initiative (CS-COUSIN).[19 20] The mission of this group is to standardise outcomes in dermatology clinical trials in order to make trial evidence more comparable and, thereby, to strengthen the quality, interpretability, and ability of systematic reviews to facilitate evidence-based decision-making in dermatology.

In this paper, we examine current practices of Cochrane systematic reviewers in relation to the use of COS in choosing outcomes, and the current views of Cochrane Review Group editors on the adoption of COS in their CRGs.

METHODS

Assessment of Cochrane reviews in relation to COS

We examined the first 100 new or updated Cochrane intervention reviews published in 2019 (dated 1st January to 8th March). We examined whether the review authors: (i) mentioned

For peer review only - http://bmjopen.bmj.com/site/about/guidelines.xhtml

using a COS to choose outcomes for the review, even if all the outcomes in the COS may not have been used; (ii) identified any problems with outcome inconsistency in the studies included in the review, and (iii) noted the need for development of a COS. We extracted information that may have been reported in any section of the completed review. One investigator (from among IJS, JJK, KH, PRW, RO, and TL) extracted information from each review and one investigator (from among MC and IJS) verified the information.

For each review, the COMET database was searched to establish whether a COS with a relevant scope (even if not an exact match) existed in 2016 or earlier, such that it might have been used to inform outcome choice at the review planning stage. For reviews where no such COS was found, the COMET database was further searched for COS published in 2017 or later, to determine whether a relevant COS may be available for consideration at a subsequent review update. Two investigators (SLG and PRW) did this independently and then compared results (data available on request).

Co-ordinating Editors of Cochrane Review Groups

We emailed the editors of all 52 CRGs (as of 2019) concerned with health interventions asking them to provide information on their involvement with COS development and their CRG's policies with regards to outcome selection for the review and for its SoF tables. They were presented with a breakdown of the total number of completed and ongoing COS for research, with references, that were within the scope of their CRG (Figure 1 summarises the number of COS by topic area).

Figure 1. Published and Ongoing Core Outcome Sets according to Cochrane Review Group

For peer review only - http://bmjopen.bmj.com/site/about/guidelines.xhtml

This list of COS was compiled from the annual update of a review of COS studies conducted by the Core Outcome Measures in Effectiveness Trials (COMET) Initiative[9-13] and was last updated in 2018.[21]

Editors were also asked about their opinions on the standardisation of outcomes within their CRG, including what they thought were up to three main advantages and three main challenges of standardising outcomes across all reviews in a particular condition covered by their CRG. They were provided with the responses from the 2012 survey for their CRG and were asked to either confirm that those earlier responses have remained the same or to identify any changes of opinion.

Expanding on the 2012 survey, we asked editors two additional questions. First, we asked if they could provide details of any examples where a COS had been considered to guide the choice of outcomes to include in a Cochrane review or a SoF table. Second, we described the aims of the CS-COUSIN initiative and asked editors for their views on the relevance and feasibility of establishing links between their CRG and an equivalent COS group.

Responses were collected between 26th February and April 30th 2019. Non-responders were contacted every 3 weeks during this period.

Analysis

The data from the assessment of Cochrane reviews were analysed descriptively.

The list of advantages and challenges of COS suggested by editors was independently reviewed by two authors (JJK and PRW) and coded using the categories identified in the

For peer review only - http://bmjopen.bmj.com/site/about/guidelines.xhtml

2012 survey, allowing new categories to be added. Discrepancies in categorization were resolved through discussion.

Patient and public involvement

Patients or the public were not involved in the design, conduct or reporting of this research.

RESULTS

Assessment of Cochrane reviews in relation to COS

The reviews examined comprised a range of health conditions (Table 1). Forty-one of the 52 current CRGs (79%) were represented. Forty reviews (40%) identified problems of inconsistency in outcomes in included studies; in five of these, the authors noted the need for development of a COS.

Table 1. Health conditions addressed in cross the 100 Cochrane Reviews

ICD-11 Code	Disease area	N (%)
12	Respiratory system	11 (11%)
16	Genitourinary system	11 (11%)
2	Neoplasms	9 (9%)
11	Circulatory system	8 (8%)
1	Infectious or parasitic diseases	5 (5%)
8	Nervous system	5 (5%)
13	Digestive system	4 (4%)
15	Musculoskeletal system	4 (4%)
9	Visual system	4 (5%)
14	Skin	3 (3%)
24	Factors influencing health status or contact with health services	3 (3%)
4	Immune system	2 (2%)
5	Endocrine, nutritional, or metabolic diseases	2 (2%)
6	Mental, behavioural, or neurodevelopmental	2 (2%)
7	Sleep-wake disorders	2 (2%)
17	Conditions related to sexual health	2 (2%)
3	Blood or blood-forming organs	1 (1%)

For peer review only - http://bmjopen.bmj.com/site/about/guidelines.xhtml

10	Ear or mastoid process	1 (1%)
19	Certain conditions originating in the perinatal period	1 (1%)
21	Symptoms, signs or clinical findings, not elsewhere classified	1 (1%)
22	Injury, poisoning, or certain other consequences of external causes	1 (1%)
23	External causes of morbidity or mortality	1 (1%)
No specific ICD-11 code	Miscellaneous	17 (17%)

Seven of 100 (7%) reviews mentioned (or cited) a COS in relation to choosing outcomes for the review. A further three reviews, while not mentioning a COS in relation to choosing outcomes for the review and not specifically recommending the use of a COS, made observations about existing COS. One review noted that several patient-important outcomes identified in an existing COS were absent from most of the review's included studies. One review acknowledged an existing COS, noting that the trials in the review continued to show inconsistency in outcomes reported, and noted that further work was needed on how the outcome domains in the COS should be measured in order to facilitate future reviews. The third review recommended that researchers should adopt the definition of a particular outcome domain of interest to the review as provided in an existing COS.

For the 90 reviews not referring to a COS, a relevant set was found to have been published in 2016 or earlier in 24. Thus, of 93 reviews not referencing a COS in relation to choosing outcomes for the review, in 27 (29%) cases this could have been possible. Of 34 reviews where a COS was available (2016 or earlier), one was used in 7 (21%). In a further 6 reviews, a relevant COS was published in 2017 or later. Thus, a relevant COS could be considered when at least 35% (33/93) of the reviews are updated.

For peer review only

For peer review only - http://bmjopen.bmj.com/site/about/guidelines.xhtml

Editors' views

Respondents

Thirty-eight (73%) of the 52 CRG editors responded to our emails, although some did not provide a response to all questions. Two-thirds of the editors (23/35, 66%) had been involved in the development of a COS, eight editors prior to the 2012 survey, and 15 since then.

In the COMET database, there were 323 published and 242 ongoing COS across all CRGs, ranging from 0 (Hypertension and Sexually Transmitted Infections) to 54 (Musculoskeletal) (Figure 1). The total number of published and ongoing COS was slightly higher for CRGs where a response was received in 2019 (median 9, IQR 5 to 16.5) compared with not (median 7, IQR 5 to 9).

Of 22 individual editors who responded in both 2012 and 2019, 64% said in 2012 that COS should be used in SoF, rising to 91% in 2019.

Cochrane Review Group Outcome Policies

Figure 2 shows the policies of CRGs for outcome selection for reviews (Figure 2a) and for SoF tables (Figure 2b); both show a reduction in a policy of authors' discretion since 2012, to 49% in 2019. Currently, 40% (14/35) of CRGs have a centralised policy for both review and SoF table outcome choice. Four CRGs (11%) indicated that outcome choice was now a negotiation between authors and the editorial team, with input from peer reviewers.

For peer review only - http://bmjopen.bmj.com/site/about/guidelines.xhtml

Figure 2. Cochrane Review Group Outcome Choice Policies (a) and SoF Table Choice Policies (b). Denominators are (n=45) and (n=35) for 2012 and 2019 respectively.

In 2019, half of the editors (18/36; 50%) thought that COS should definitely be considered in the process of choosing outcomes for a SoF, whilst a further 13 (36%) editors thought that COS could possibly be used in some circumstances if relevant to the specific review and if the COS was developed using a 'quality' consensus approach. Two (6%) editors thought that COS should not be used in the SoF tables because they can lead to the inclusion of unnecessary outcomes. One editor did not think that the inclusion of COS in SoF tables was relevant, because they did not think COS were used in their topic area. Two editors were unsure, because they were aware that many COS contain more than the seven outcomes allowed for SoF tables.

Advantages and Challenges of Standardising Outcomes in Cochrane Reviews

For both the advantages and challenges associated with standardising outcomes across reviews (Table 2), the three most commonly listed were the same as in the 2012 survey. Just under half of the editors consider an advantage of using a COS to be that the outcomes are likely to be more appropriate.

Table 2. Views of Cochrane Editors on standardising outcomes across all reviews for a particular condition

**Advantages**	**2012 n/40 (%*)**	**2019 n/32 (%*)**
Advantage for a systematic review/meta-analysis	39 (98)	27 (84)
Improves interpretation/guidance	19 (48)	7 (22)
Outcome likely to be more appropriate	16 (40)	10 (45)
Advantage for the design of a new study	13 (33)	5 (16)

For peer review only - http://bmjopen.bmj.com/site/about/guidelines.xhtml

Improves something about the outcome itself (e.g. simplifies the reporting)	6 (15)	0 (0)
Reduces outcome reporting bias	6 (15)	1 (3)
Reduces resource requirement (e.g. time to review)	1 (3)	1 (3)
**Challenges**	**2012 n/42 (%*)**	**2019 n/36 (%*)**
Development of a COS	23 (55)	8 (22)
Something about scope	21 (50)	28 (78)
How to persuade authors/trialists/industry to implement	20 (48)	14 (39)
'How' to measure once the 'what' has been decided	11 (26)	3 (8)
Important outcomes not currently being measured	2 (5)	1 (3)
Resource to develop	2 (5)	3 (8)
Updating process	2 (5)	2 (6)
Conflict of interest	1 (1)	0 (0)
Limits authors	0 (2)	3 (8)

*Percentages represent the number of Cochrane Review Group Co-ordinating Editors who mentioned each advantage/challenge

One notable difference was a shift from the most frequently-listed challenge being COS development in 2012 (55% in 2012, falling to third place on 22% in 2019) to deciding when a COS should be applied (in relation to the scope of the review and the scope of the COS) in 2019 (78%). One new challenge identified in 2019 by three editors (8%) was the perception that the use of a COS may limit review authors due to a lack of flexibility in outcome choice.

Links between CRG, COS developers and Guidance

Eight editors identified cases where the outcome choice in at least one systematic review in their group had been informed by a COS. The majority (22/26, 85%) of editors thought that links between their CRG and COS developers in their field would be a good idea in principle, whilst four could see no benefit. In addition to the Cochrane Skin Group, three CRGs had already established such links: the Cochrane Musculoskeletal Group with the Outcome Measures in Rheumatology (OMERACT) Initiative,[22] the Cochrane Kidney and Transplant Group with the Standardising Outcomes in Nephrology (SONG) Initiative,[23] and the

For peer review only - http://bmjopen.bmj.com/site/about/guidelines.xhtml

Cochrane Pregnancy and Childbirth Group with the Core Outcomes in Women's and Newborn Health (CROWN) Initiative.[24]

The major perceived barriers to establishing links between CRGs and COS developers were related to lack of resources, mainly lack of time (mentioned nine times) and funding to co-ordinate (mentioned five times). Conflicts of interest, the potential for poor communication between members, and lack of knowledge on how to manage such links were also quoted as potential barriers to implementation.

CONCLUSIONS

Our study has demonstrated that Cochrane systematic reviewers note widespread outcome inconsistency across studies. We found that almost all CRG editors think that COS should definitely or possibly be used in SoF tables, and that reviewers are beginning to use COS to inform their choice of outcomes.

We recorded increased involvement of CRG editors in COS development studies since 2012. The COS-STAD (Core Outcome Set-STAndards for Development) guidance identifies three stakeholder groups as the minimum for input into the development of a COS: patients or their representatives, healthcare professionals, and those who will use the COS in research.[14] Consensus was not quite achieved that a fourth group, those who will use the research that should use the COS (e.g., systematic reviewers, guideline developers, policy makers, regulatory agencies), should always be involved. COS-STAD participants may have wanted to avoid setting a minimum standard when there was limited experience of engaging with these particular stakeholder groups. If the involvement of Cochrane

For peer review only - http://bmjopen.bmj.com/site/about/guidelines.xhtml

reviewers in COS development studies continues to increase, it would seem appropriate to review the standard in due course.

The majority of editors were in favour of cooperation between COS developers and their CRG, however the most frequently mentioned potential barriers, such as lack of time, resources, and funding, will need to be addressed to achieve this. The affiliation of a COS development initiative to a Cochrane Review Group, as with CS-COUSIN in dermatology,[19 20] could offer easier access to information about outcomes previously measured in trials, and to Cochrane's international network of consumers, healthcare professionals, and researchers in the field. Additionally, close interaction between COS development and CRGs should support COS implementation. For the dermatology outcome research community, the formal affiliation of COS development groups to Cochrane is considered an important advantage. This strategy has led to the affiliation of 15 COS groups to CS-COUSIN since its initiation in 2015.[19 20]

We achieved a 73% response rate from CRG Co-ordinating Editors. Whilst this rate is acceptable, it is conceivable that non-response bias may have arisen as a result of a lack of interest or awareness about COS among editors not responding.

Using a given COS for a given systematic review involves, at the least, an assessment of the appropriateness of the topic, scope, stakeholder representation, and currency of the COS. We recommend that, when a potentially appropriate COS exists for a given systematic review, the authors should either use it to inform their choice of outcomes or justify their reason for not using it. The COMET database of COS (available online at http://www.comet-

For peer review only - http://bmjopen.bmj.com/site/about/guidelines.xhtml

initiative.org/), is free, searchable, and covers a range of health fields. In addition, authors of Cochrane reviews, especially those that identify outcome inconsistency in included studies, should take the opportunity to move their respective fields forward by explicitly noting the need for COS, recommending COS development, and participating in the COS development process.

The use of COS for a systematic review in turn may influence trialists to use that same COS to ensure that their trials can contribute to subsequent reviews and meta-analyses. Given the continued problem of outcome inconsistency in studies documented in Cochrane reviews, and the fact that four CRGs have already established links to COS development groups, this topic could be a potential area for strategic development of Cochrane. Such greater uptake of COS across the healthcare research ecosystem will help towards improving health through improving healthcare.

Acknowledgements: We thank all the Cochrane Review Group Co-ordinating editors who responded to our questions.

Funding: This work was supported by an MRC Hub for Trials Methodology Research Network studentship (grant reference MR/L004933/1-Q30); a National Institute for Health Research (NIHR) Senior Investigator Award (NF-SI_0513-10025); and the MRC Trials Methodology Research Partnership (grant reference MR/S014357/1). The views expressed in this article are those of the authors and not necessarily those of the NIHR, or the Department of Health and Social Care.

For peer review only - http://bmjopen.bmj.com/site/about/guidelines.xhtml

Competing Interests: PRW and MC are members of the COMET (Core Outcome Measures in Effectiveness Trials) Management Group. JJK and JS are members of the CS-COUSIN (Cochrane Skin – Core Outcome Set Initiative) methods group. The remaining authors declare no competing interests.

Authors' Contributions: PRW conceived the idea for the manuscript. JJK, MC, JS and PRW developed the questions for the Cochrane Review Group Co-ordinating Editors. The list of the total number of completed and ongoing COS with references for each CRG was produced by SG. The requests to the editors in 2019 were administered by JJK. Their responses were analysed by JJK in collaboration with PRW. The review of Cochrane reviews was designed by IS and PRW. The data were extracted by RdeAO, KH, JJK, TL, IS and PRW, confirmed by MC, and analysed by IS. The assessment of relevance of published COS was undertaken by SG and PRW. PRW, JJK and IS prepared the initial manuscript. All authors commented on and approved the final manuscript before submission.

Data Sharing Statement: All data are available from the corresponding author on reasonable request.

For peer review only - http://bmjopen.bmj.com/site/about/guidelines.xhtml

References

1. Clarke M, Williamson PR. Core outcome sets and systematic reviews. *Systematic Reviews* 2016;5:11.
2. Kirkham JJ, Dwan KM, Altman DG, et al. The impact of outcome reporting bias in randomised controlled trials on a cohort of systematic reviews. *BMJ* 2010;340:c365.
3. Berglas S, Jutai L, MacKean G, et al. Patients' perspectives can be integrated in health technology assessments: an exploratory analysis of CADTH Common Drug Review. *Res Involv Engagem* 2016;2:21.
4. Glasziou P, Altman DG, Bossuyt P, et al. Reducing waste from incomplete or unusable reports of biomedical research. *The Lancet* 2014;383(9913):267-76.
5. Saldanha IJ, Lindsley K, Do DV, et al. Comparison of Clinical Trial and Systematic Review Outcomes for the 4 Most Prevalent Eye Diseases. *JAMA Ophthalmol* 2017;135(9):933-40.
6. Saldanha IJ, Li T, Yang C, et al. Clinical trials and systematic reviews addressing similar interventions for the same condition do not consider similar outcomes to be important: A case study in HIV/AIDS. *Journal of Clinical Epidemiology* 2017;84:85-94.
7. Kirkham JJ, Gargon E, Clarke M, et al. Can a core outcome set improve the quality of systematic reviews? – a survey of the Co-ordinating Editors of Cochrane review groups. *Trials* 2013;14:21.
8. COMET Initiative Database Search 2019 [Available from: http://www.comet-initiative.org/studies/search accessed cited 23 April 2019.
9. Gargon E, Gurung B, Medley N, et al. Choosing important health outcomes for comparative effectiveness research: a systematic review. *PLoS One* 2014;9(6):e99111.
10. Gorst SL, Gargon E, Clarke M, et al. Choosing Important Health Outcomes for Comparative Effectiveness Research: An Updated Review and User Survey. *PLoS One* 2016;11(1):e0146444.
11. Gorst SL, Gargon E, Clarke M, et al. Choosing Important Health Outcomes for Comparative Effectiveness Research: An Updated Review and Identification of Gaps. *PLoS One* 2016;11(12):e0168403.
12. Davis K, Gorst SL, Harman N, et al. Choosing important health outcomes for comparative effectiveness research: An updated systematic review and involvement of low and middle income countries. *PLoS One* 2018;13(2):e0190695.
13. Gargon E, Gorst SL, Harman NL, et al. Choosing important health outcomes for comparative effectiveness research: 4th annual update to a systematic review of core outcome sets for research. *PLoS One* 2018;13(12):e0209869.
14. Kirkham JJ, Davis K, Altman DG, et al. Core Outcome Set-STAndards for Development: The COS-STAD recommendations. *PLoS Med* 2017;14(11):e1002447.
15. Kirkham JJ, Gorst S, Altman DG, et al. Core Outcome Set-STAndards for Reporting: The COS-STAR Statement. *PLoS Med* 2016;13(10):e1002148.
16. Kirkham JJ, Gorst SL, Altman DG, et al. Core Outcome Set-STAndardised Protocol Items: The COS-STAP Statement. *Trials* 2019;20:116.
17. Williamson PR, Altman DG, Bagley H, et al. The COMET Handbook: version 1.0. *Trials* 2017;18(Suppl 3):280.
18. Higgins JPT, Thomas J, Chandler J, et al., editors. *Cochrane Handbook for Systematic Reviews of Interventions. 2nd Edition*. Chichester (UK): John Wiley & Sons, 2019.
19. Cochrane Skin: Core Outcome Set Initiative (CS-COUSIN) 2019 [Available from: http://cs-cousin.org/ accessed cited 23 April 2019.
20. Prinsen CAC, Spuls PI, Kottner J, et al. Navigating the landscape of core outcome set development in dermatology. *J Am Acad Dermatol* 2019;81(1):297-305.
21. Gargon E, Gorst SL, Williamson PR. The use of automated article ranking in the fifth annual update to a systematic review of core outcome sets for research. *Submitted* 2019

For peer review only - http://bmjopen.bmj.com/site/about/guidelines.xhtml

22. OMERACT Initiative 2019 [Available from: https://omeract.org/ accessed cited 23 April 2019.
23. SONG Initiative 2019 [Available from: https://songinitiative.org/ accessed cited 23 April 2019.
24. CROWN Initiative 2019 [Available from: http://www.crown-initiative.org/ accessed cited 23 April 2019.

For peer review only

For peer review only - http://bmjopen.bmj.com/site/about/guidelines.xhtml

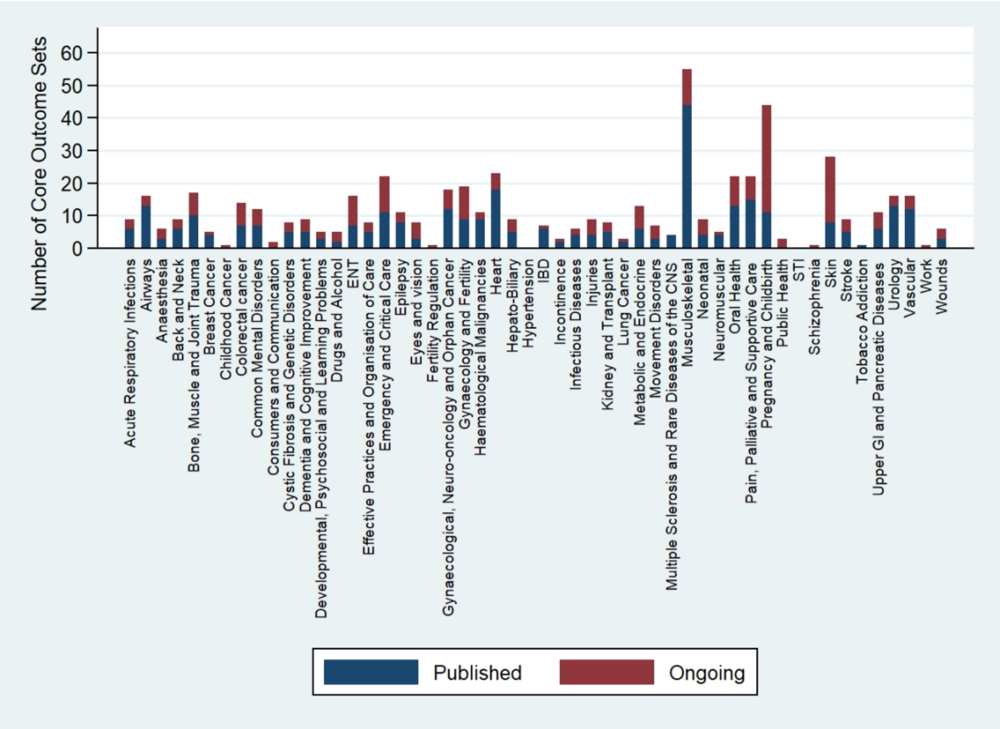

108x78mm (300 x 300 DPI)

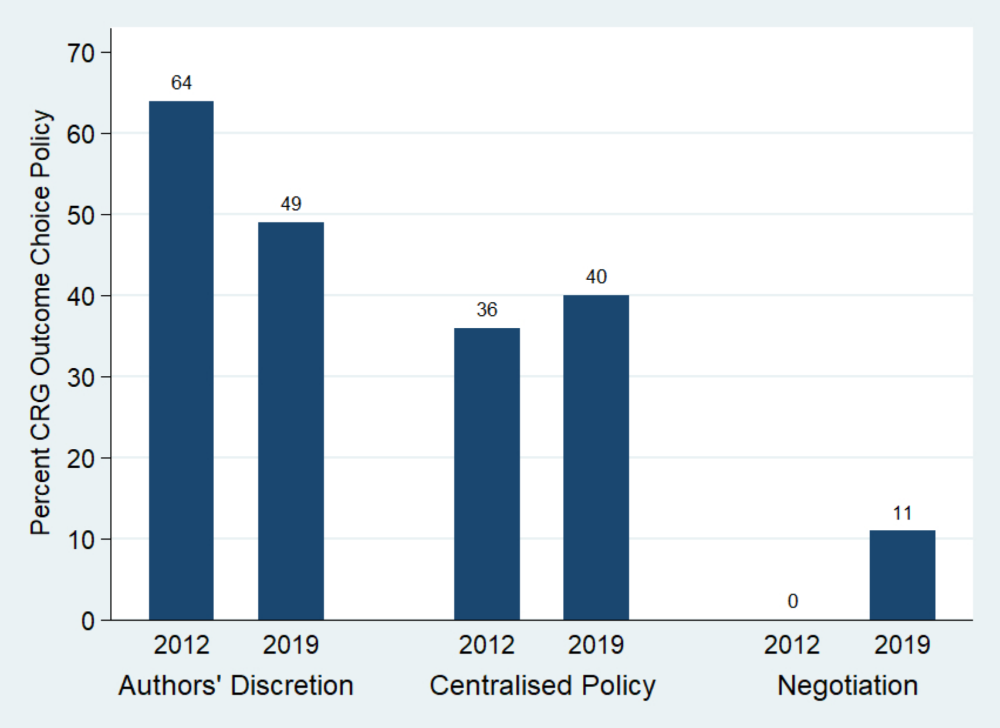

72x52mm (300 x 300 DPI)

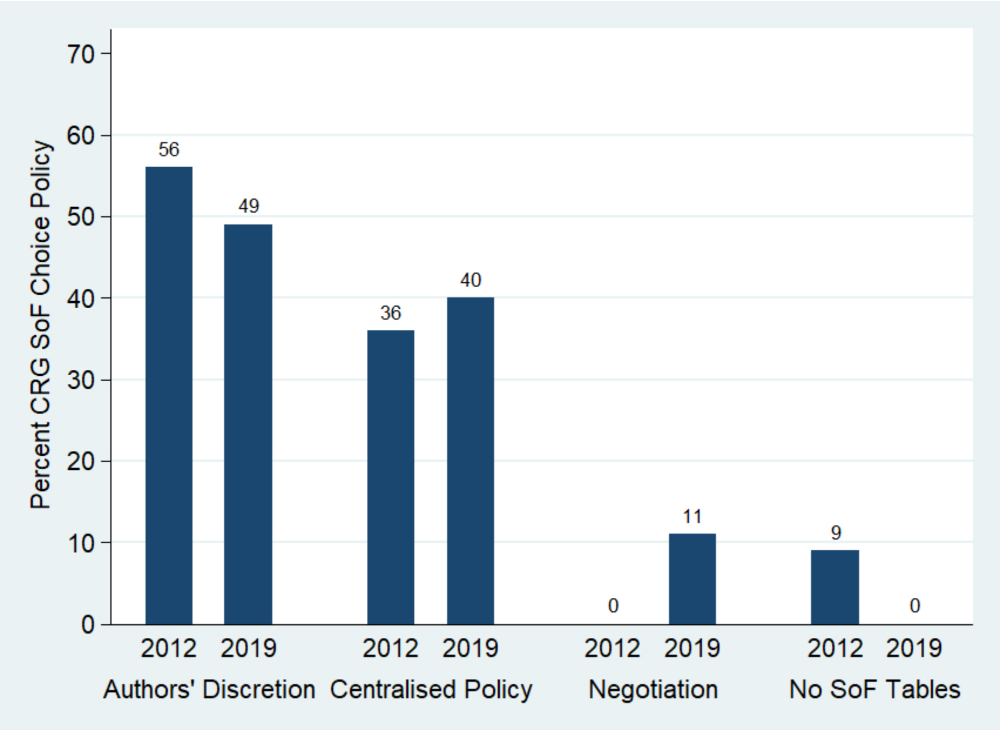

72x52mm (300 x 300 DPI)

BMJ Open

Assessing the relevance and uptake of core outcome sets (an agreed minimum collection of outcomes to measure in research studies) in Cochrane systematic reviews: a review

Journal:	*BMJ Open*
Manuscript ID	bmjopen-2019-036562.R1
Article Type:	Original research
Date Submitted by the Author:	22-May-2020
Complete List of Authors:	Williamson, Paula; University of Liverpool,<br>de Ávila Oliveira, Ricardo; Universidade Federal de Uberlândia<br>Clarke, Mike; Queen's University Belfast, Northern Ireland Methodology Hub<br>Gorst, Sarah L.; University of Liverpool<br>Hughes, Karen; University of Liverpool<br>Kirkham, Jamie J.; University of Liverpool, Biostatistics<br>Li, Tianjing; University of Colorado<br>Saldanha, Ian; Brown University<br>Schmitt, Jochen; Technische Universität Dresden, Department of Dermatology
**Primary Subject Heading**:	Health services research
Secondary Subject Heading:	Research methods
Keywords:	STATISTICS & RESEARCH METHODS, Clinical trials < THERAPEUTICS, GENERAL MEDICINE (see Internal Medicine)

SCHOLARONE™
Manuscripts

I, the Submitting Author has the right to grant and does grant on behalf of all authors of the Work (as defined in the below author licence), an exclusive licence and/or a non-exclusive licence for contributions from authors who are: i) UK Crown employees; ii) where BMJ has agreed a CC-BY licence shall apply, and/or iii) in accordance with the terms applicable for US Federal Government officers or employees acting as part of their official duties; on a worldwide, perpetual, irrevocable, royalty-free basis to BMJ Publishing Group Ltd ("BMJ") its licensees and where the relevant Journal is co-owned by BMJ to the co-owners of the Journal, to publish the Work in this journal and any other BMJ products and to exploit all rights, as set out in our licence.

The Submitting Author accepts and understands that any supply made under these terms is made by BMJ to the Submitting Author unless you are acting as an employee on behalf of your employer or a postgraduate student of an affiliated institution which is paying any applicable article publishing charge ("APC") for Open Access articles. Where the Submitting Author wishes to make the Work available on an Open Access basis (and intends to pay the relevant APC), the terms of reuse of such Open Access shall be governed by a Creative Commons licence – details of these licences and which Creative Commons licence will apply to this Work are set out in our licence referred to above.

Other than as permitted in any relevant BMJ Author's Self Archiving Policies, I confirm this Work has not been accepted for publication elsewhere, is not being considered for publication elsewhere and does not duplicate material already published. I confirm all authors consent to publication of this Work and authorise the granting of this licence.

For peer review only

Assessing the relevance and uptake of core outcome sets (an agreed minimum collection of outcomes to measure in research studies) in Cochrane systematic reviews: a review

Paula R Williamson[a*]

Ricardo de Ávila Oliveira[b]

Mike Clarke[c]

Sarah L Gorst[a]

Karen Hughes[a]

Jamie J Kirkham[d]

Tianjing Li[e]

Ian J Saldanha[f]

Jochen Schmitt[g]

[a] MRC North West Hub for Trials Methodology Research, University of Liverpool and member of Liverpool Health Partners, Liverpool, United Kingdom

[b] DECIR Faculdade de Medicina, Universidade Federal de Uberlândia, Uberlândia, Brazil

[c] Northern Ireland Methodology Hub, Centre for Public Health, Queen's University Belfast, Belfast, United Kingdom

[d] Centre for Biostatistics, Manchester Academic Health Science Centre, University of Manchester, Manchester, United Kingdom

[e] Department of Ophthalmology, University of Colorado Denver, Aurora, Colorado, USA

[f] Center for Evidence Synthesis in Health, Brown University School of Public Health, Providence, Rhode Island, USA

For peer review only - http://bmjopen.bmj.com/site/about/guidelines.xhtml

[g] Center for Evidence-based Healthcare, Medizinische Fakultät, Technische Univesität Dresden, Dresden, Germany

[*]**Corresponding Author:**

Professor Paula Williamson

Department of Biostatistics

University of Liverpool

Block F Waterhouse Building, 1-5 Brownlow Street, Liverpool, L69 3GL, United Kingdom

Email: prw@liv.ac.uk

Tel: +44 (0) 151 794 9758

Word Count: 3340

For peer review only

For peer review only - http://bmjopen.bmj.com/site/about/guidelines.xhtml

ABSTRACT

Objectives: A core outcome set (COS) is an agreed standardised minimum collection of outcomes that should be measured and reported in research in a specific area of health. This study examines the use of existing COS to inform the choice of outcomes in Cochrane systematic reviews, and investigates the views of the Co-ordinating Editors of Cochrane Review Groups on this topic.

Methods: A cohort of 100 recently published or updated Cochrane systematic reviews were assessed for reference to a COS being used to inform the choice of review outcomes. Existing COS, published two or more years before the review publication, were then identified to assess how often a reviewer could have used a relevant COS if it was available. We asked 52 Cochrane Review Group (CRG) Co-ordinating Editors about their involvement in COS development, how outcomes are selected for reviews in their CRG, and their views of the advantages and challenges surrounding the standardisation of outcomes within their CRG.

Results: In the cohort of reviews from 2019, 40% (40/100) of reviewers noted problems due to outcome inconsistency across the included studies. In 7% (7/100), a COS was referenced in relation to the choice of outcomes for the review. Relevant existing COS could be considered for a review update in 35% of the others (33/93). Most responding editors (31/36, 86%) thought that COS should definitely or possibly be used to inform the choice of outcomes in a review.

For peer review only - http://bmjopen.bmj.com/site/about/guidelines.xhtml

Conclusions: Systematic reviewers are continuing to note outcome heterogeneity but are starting to use COS to inform their reviews. There is potential for greater uptake of COS in Cochrane reviews.

Keywords: Cochrane Review Group; Co-ordinating Editor; core outcome set; summary of findings; systematic reviews

STRENGTHS AND LIMITATIONS OF THIS STUDY

- A sample of 100 Cochrane systematic reviews that were published for the first time or updated in 2019 were assessed.
- Two investigators independently assessed the relevance of a core outcome set (COS) to each systematic review.
- An assumption was made that if a COS had been published two or more years before the publication of the systematic review, it would have been available to the reviewers to adopt.
- Co-ordinating Editors for all Cochrane Review Groups were asked for their views on the relevance of COS to systematic reviews.
- The research was undertaken before the release of version 6 of the Cochrane Handbook for Systematic Reviews of Interventions, which recommends that Cochrane reviewers should consider using a COS.

For peer review only - http://bmjopen.bmj.com/site/about/guidelines.xhtml

BACKGROUND

Systematic reviews of the effects of healthcare interventions summarise the available evidence and are used for decision-making. Inconsistencies in the outcomes that are measured and reported across included studies can mean that it is not possible for all studies to be included in a meta-analysis however, a form of research waste. In addition, the selective reporting of outcomes within a study based on the results, known as outcome reporting bias, can affect the robustness of the conclusions of a systematic review.[1 2]

Furthermore, there is increasing awareness that differences exist between the outcomes measured in trials and outcomes that patients consider important. For example, only 50% of the outcomes that patients said matter to them were captured in the primary studies reviewed by the Canadian Agency for Drugs and Technologies in Health (CADTH) Common Drug Review.[3] Such problems also contribute to research waste.[4]

One solution to these problems with the choice of outcomes is for trialists in a particular topic area to measure and report a core outcome set (COS), which would then be used in reviews addressing that same topic area. A COS is an agreed standardised collection of outcomes that should be measured and reported in a specific area of health. It does not preclude the inclusion of additional outcomes, but represents the minimum for all research studies in the topic area. The scope of a COS refers to the specific area of health or health care of interest to which the COS is to be applied, defined in terms of the health condition, population, and healthcare interventions covered. Thus within a particular topic area there may be multiple COS, depending on whether a COS is developed for different populations, for example adults and children, or localised and advanced disease. A COS may be

For peer review only - http://bmjopen.bmj.com/site/about/guidelines.xhtml

developed to apply to any intervention for that condition, or it may be for specific types, for example surgery.

Minimum standards have been developed for determining COS.[5] According to these standards, as a minimum, the stakeholders to be included in determining a core outcome set should be those with lived experience of the condition, health professionals caring for those with lived experience, and those undertaking research in the condition (REF). The benefits of using a COS are to increase consistency across trials, resulting in a major reduction in selective reporting and maximising the potential for a trial to contribute to meta-analyses of these key outcomes.[6] Of crucial importance is that given the expectations of relevant stakeholder involvement, the use of a COS should mean trialists are much more likely to measure appropriate outcomes that are relevant to patients.

An additional issue in the choice of outcomes for a systematic review relates to clinical trialists and systematic reviewers sometimes being interested in different types of outcomes.[7 8] Clarke and Williamson have argued for greater involvement of systematic reviewers in the development and implementation of COS[1], suggesting this might help with planning of reviews and ensuring that reviews identify data for outcomes of interest.

Cochrane reviews are rigorous systematic reviews conducted under the auspices of Cochrane, a global and independent volunteer organisation with the primary goal of conducting evidence syntheses in health. Cochrane is organised operationally into Cochrane Review Groups (CRGs), comprised of an editorial team, information specialists, and authors responsible for reviews within distinct clinical and public health areas. For specific reviews,

For peer review only - http://bmjopen.bmj.com/site/about/guidelines.xhtml

decisions need to be made regarding outcomes that will be included in the review as well as outcomes that will be prioritised for inclusion in the Summary of Findings (SoF) tables. SoF tables are intended to provide consumers of the review with the key findings of the review.

Each CRG is led by a Co-ordinating Editor who oversee the production and maintenance of Cochrane reviews. In a 2012 survey of CRG Co-ordinating Editors (hereafter "editors"), the majority (33/45, 73%) thought that COS should routinely be used in the SoF tables.[9] At that time, a third of the editors (14/45, 31%) reported personal involvement in the development of a COS.

The awareness and development of COS have substantially increased over recent years, with more than 100 new COS published between 2012 and 2019, and at least 200 more whose development are ongoing.[10-16] A series of guidelines have been published to improve the design and reporting of COS studies.[17-20] The relevance of COS to defining review questions and planning the review, as well as the inclusion of patient-centred outcomes within COS, is acknowledged in the 2019 version of the Cochrane Handbook, which states, "where available, established sets of core outcomes should be used".[21] One notable link between COS development and Cochrane is the work of the Cochrane Skin Group on the Cochrane Skin Core Outcome Set Initiative (CS-COUSIN).[22 23] The mission of this group is to standardise outcomes in dermatology clinical trials in order to make trial evidence more comparable and, thereby, to strengthen the quality, interpretability, and ability of systematic reviews to facilitate evidence-based decision-making in dermatology.

For peer review only - http://bmjopen.bmj.com/site/about/guidelines.xhtml

In this paper, we examine current practices of Cochrane systematic reviewers in relation to the use of COS in choosing outcomes, and the current views of Cochrane Review Group editors on the adoption of COS in their CRGs.

METHODS

Assessment of Cochrane reviews in relation to COS

Although a formal protocol was not developed for this study, the following approach was agreed amongst the research team in advance. We examined the first 100 new or updated Cochrane intervention reviews published in 2019 (dated 1st January to 8th March). We chose to restrict to reviews published in 2019 in order to determine contemporary practices regarding outcome selection in Cochrane reviews. The sample size was a pragmatic choice, to give a reasonable number of reviews that we could assess in a reasonable period of time.

We examined whether, in the completed version of the review, the review authors: (i) mentioned using a COS to choose outcomes for the review, even if all the outcomes in the COS may not have been used; (ii) identified any problems with outcome inconsistency across the studies included in the review, and (iii) noted the need for development of a COS. We extracted information that may have been reported in any section of the completed review. One investigator (from among IJS, JJK, KH, PRW, RdeAO, and TL) extracted information from each review and one investigator (from among MC and IJS) verified the information.

For each review, we searched the COMET database to establish whether a COS with a relevant scope (even if not an exact match) had been published by 31 December 2016, such

For peer review only - http://bmjopen.bmj.com/site/about/guidelines.xhtml

that it might have been available to inform outcome choice at the planning stage for reviews that were published more than two years later. For reviews where no such COS was found, the COMET database was also searched for COS published since 1 January 2017, to determine whether a relevant COS may be available for consideration when the Cochrane review is updated. Two investigators (SLG and PRW) assessed the potential relevance of identified COS independently and then compared their assessments, following an approach used previously (data available on request).[24]

Co-ordinating Editors of Cochrane Review Groups

Consistent with the approach used in our 2012 survey[7], we emailed the editors of all 52 CRGs (as of 2019) requesting them to provide information on their involvement with COS development and their CRG policy with regards to outcome selection for reviews and prioritisation of outcomes for associated SoF tables. They were presented with a breakdown of the total number of completed and ongoing COS for research, with references, that were within the scope of their CRG (Figure 1 summarises the number of COS by topic area).

Editors were also asked about their opinions on the standardisation of outcomes within their CRG, including what they thought were up to three main advantages and three main challenges of standardising outcomes across all reviews in a particular condition covered by their CRG. They were provided with the responses from the 2012 survey for their CRG and were asked to either confirm that those earlier responses remained the same or to identify any changes of opinion.

For peer review only - http://bmjopen.bmj.com/site/about/guidelines.xhtml

Expanding on our 2012 survey, we requested them to provide details of any examples where a COS had been considered to guide the choice of outcomes to include in a Cochrane review or a SoF table. Second, we described the aims of the CS-COUSIN initiative and asked editors for their views on the relevance and feasibility of establishing links between their CRG and an equivalent COS group.

Responses were collected between 26th February and April 30th 2019. Non-responders were contacted every 3 weeks during this period.

Analysis

The data from the assessment of Cochrane reviews were analysed descriptively. The lists of advantages and challenges of COS suggested by editors was independently reviewed by two authors (JJK and PRW) and coded using the categories identified in the 2012 survey, allowing new categories to be added. Discrepancies in categorisation were resolved through discussion.

Patient and public involvement

Patients or the public were not involved in the design, conduct or reporting of this research.

RESULTS

Assessment of Cochrane reviews in relation to use of COS to inform outcome choice

The reviews assessed addressed a range of health conditions (Table 1). Forty-one of the 52 current CRGs (79%) were represented. Forty reviews (40%) identified problems of

For peer review only

For peer review only - http://bmjopen.bmj.com/site/about/guidelines.xhtml

inconsistency in outcomes in included studies; in five of these, the authors also explicitly noted the need for development of a COS.

Table 1. Health conditions addressed in the 100 Cochrane Reviews assessed in relation to use of COS

ICD-11 Code	Disease area	N (%)
12	Respiratory system	11 (11%)
16	Genitourinary system	11 (11%)
2	Neoplasms	9 (9%)
11	Circulatory system	8 (8%)
1	Infectious or parasitic diseases	5 (5%)
8	Nervous system	5 (5%)
13	Digestive system	4 (4%)
15	Musculoskeletal system	4 (4%)
9	Visual system	4 (5%)
14	Skin	3 (3%)
24	Factors influencing health status or contact with health services	3 (3%)
4	Immune system	2 (2%)
5	Endocrine, nutritional, or metabolic diseases	2 (2%)
6	Mental, behavioural, or neurodevelopmental	2 (2%)
7	Sleep-wake disorders	2 (2%)
17	Conditions related to sexual health	2 (2%)
3	Blood or blood-forming organs	1 (1%)
10	Ear or mastoid process	1 (1%)
19	Certain conditions originating in the perinatal period	1 (1%)
21	Symptoms, signs or clinical findings, not elsewhere classified	1 (1%)
22	Injury, poisoning, or certain other consequences of external causes	1 (1%)
23	External causes of morbidity or mortality	1 (1%)
No specific ICD-11 code	Miscellaneous	17 (17%)

Seven of 100 (7%) reviews mentioned (or cited) a COS in relation to choosing outcomes for the review. A further three reviews, while not mentioning a COS in relation to choosing outcomes for the review and not specifically recommending the use of a COS, made observations about existing COS. One review noted that several patient-important

For peer review only - http://bmjopen.bmj.com/site/about/guidelines.xhtml

outcomes identified in an existing COS were absent from most of the review's included studies. One review acknowledged an existing COS, noting that the trials in the review continued to report inconsistent outcomes, and noted that further work was needed on how the outcome domains in the COS should be measured to facilitate future reviews. The third review recommended that researchers should adopt the definition of a particular outcome domain of interest to the review as provided in an existing COS.

For the 90 reviews not referring to a COS, a relevant COS was found to have been published in 2016 or earlier in 24. Thus, of 93 reviews not referencing a COS in relation to choosing outcomes for the review, in 27 (29%) cases, this could have been possible.

In summary, of the 100 reviews, a COS from 2016 or earlier was available for 34 (34%). Of those 34 reviews, a COS was used in 7 (21%). In a further six reviews, a relevant COS was published in 2017 or later. Thus, a relevant COS could be considered during the updating of at least 35% (33/93) of the reviews that have not already used a COS.

Editors' views

Thirty-eight (73%) of the 52 CRG editors responded to our emails, although some did not provide a response to all questions. Two-thirds of the editors (23/35, 66%) had been involved in the development of a COS, eight editors prior to the 2012 survey, and 15 since then.

As of 23rd April 2019, the COMET database contained 323 published and 242 ongoing COS across all CRGs, ranging from 0 (Hypertension and Sexually Transmitted Infections) to 54

For peer review only - http://bmjopen.bmj.com/site/about/guidelines.xhtml

(Musculoskeletal) (Figure 1). The total number of published and ongoing COS was slightly higher for CRGs whose editors responded in 2019 (median 9, interquartile range (IQR) 5 to 16.5) compared with not (median 7, IQR 5 to 9).

Of 22 editors who responded in both 2012 and 2019, 64% noted in 2012 that COS should be used in SoF; this percentage rose to 91% in 2019.

Cochrane Review Group Outcome Policies

Figures 2 and 3 show the policies of CRGs for outcome selection for reviews (Figure 2) and for SoF tables (Figure 3); both show a reduction in a policy of authors' discretion since 2012, to 49% in 2019. Currently, 40% (14/35) of CRGs have a centralised policy for both review and SoF table outcome choice. Four editors (11%) indicated that outcome choice was now a negotiation between authors and the editorial team, with input from peer reviewers.

In 2019, half of the editors (18/36; 50%) thought that COS should definitely be considered in the process of choosing outcomes for a SoF, whilst a further 13 (36%) editors thought that COS could possibly be used in some circumstances if relevant to the specific review and if the COS was developed using a 'quality' consensus approach. Two (6%) editors thought that COS should not be used in the SoF tables because they can lead to the inclusion of unnecessary outcomes. One editor did not think that the inclusion of COS in SoF tables was relevant, because they did not think COS were used in their topic area. Two editors were unsure because they were aware that many COS contain more than the maximum of seven outcomes that Cochrane allows for SoF tables.

For peer review only - http://bmjopen.bmj.com/site/about/guidelines.xhtml

Advantages and Challenges of Standardising Outcomes in Cochrane Reviews

For both the advantages and challenges associated with standardising outcomes across reviews (Table 2), the three most commonly listed were the same as in the 2012 survey. Just under half of the editors consider an advantage of using a COS to be that the outcomes are likely to be more appropriate.

Table 2. Views of Cochrane Editors on standardising outcomes across all reviews for a particular condition

**Advantages**	**2012 n/40 (%*)**	**2019 n/32 (%*)**
Advantage for a systematic review/meta-analysis	39 (98)	27 (84)
Improves interpretation/guidance	19 (48)	7 (22)
Outcome likely to be more appropriate	16 (40)	10 (45)
Advantage for the design of a new study	13 (33)	5 (16)
Improves something about the outcome itself (e.g. simplifies the reporting)	6 (15)	0 (0)
Reduces outcome reporting bias	6 (15)	1 (3)
Reduces resource requirement (e.g. time to review)	1 (3)	1 (3)
**Challenges**	**2012 n/42 (%*)**	**2019 n/36 (%*)**
Development of a COS	23 (55)	8 (22)
Something about scope	21 (50)	28 (78)
How to persuade authors/trialists/industry to implement	20 (48)	14 (39)
'How' to measure once the 'what' has been decided	11 (26)	3 (8)
Important outcomes not currently being measured	2 (5)	1 (3)
Resource to develop	2 (5)	3 (8)
Updating process	2 (5)	2 (6)
Conflict of interest	1 (1)	0 (0)
Limits authors	0 (2)	3 (8)

*Percentages represent the number of Cochrane Review Group Co-ordinating Editors who mentioned each advantage/challenge

One notable difference from 2012 was a shift from the most frequently-listed challenge being COS development (55% in 2012, falling to third place on 22% in 2019) to deciding when a COS should be applied (in relation to the scope of the review and the scope of the COS) in 2019 (78%). One new challenge identified in 2019 by three editors (8%) was the

For peer review only - http://bmjopen.bmj.com/site/about/guidelines.xhtml

perception that the use of a COS may limit review authors due to a lack of flexibility in outcome choice.

Links between CRG, COS developers and Guidance

Eight editors identified cases where the outcome choice in at least one systematic review in their group had been informed by a COS. The majority (22/26, 85%) of editors thought that links between their CRG and COS developers in their field would be a good idea in principle, whilst four could see no benefit. In addition to the Cochrane Skin Group, three CRGs had already established such links: the Cochrane Musculoskeletal Group with the Outcome Measures in Rheumatology (OMERACT) Initiative,[25] the Cochrane Kidney and Transplant Group with the Standardising Outcomes in Nephrology (SONG) Initiative,[26] and the Cochrane Pregnancy and Childbirth Group with the Core Outcomes in Women's and Newborn Health (CROWN) Initiative.[27]

The major perceived barriers to establishing links between CRGs and COS developers were related to lack of resources, mainly lack of time (mentioned nine times) and funding to co-ordinate (mentioned five times). Conflicts of interest, the potential for poor communication between members, and lack of knowledge on how to manage such links were also quoted as potential barriers to implementation.

CONCLUSIONS

Our study has demonstrated that Cochrane systematic reviewers note widespread outcome inconsistency across studies. We found that almost all CRG editors think that COS should

For peer review only - http://bmjopen.bmj.com/site/about/guidelines.xhtml

definitely or possibly be used in SoF tables, and that reviewers are beginning to use COS to inform their choice of outcomes.

We have documented an increased level of involvement of CRG editors in COS development studies since 2012. The COS-STAD (Core Outcome Set-STAndards for Development) guidance identifies three stakeholder groups as the minimum for input into the development of a COS: patients or their representatives, healthcare professionals, and those who will use the COS in research.[17] Consensus was not quite achieved that a fourth group, those who will use the research that should use the COS (e.g., systematic reviewers, guideline developers, policy makers, regulatory agencies), should always be involved. COS-STAD participants may have wanted to avoid setting a minimum standard when there was limited experience of engaging with these particular stakeholder groups. If the involvement of Cochrane reviewers in COS development studies continues to increase, it would seem appropriate to review the standard in due course.

The majority of editors were in favour of cooperation between their CRG and COS developers in their field, however the most frequently mentioned potential barriers, such as lack of time, resources, and funding, will need to be addressed to achieve this. The affiliation of a COS development initiative to a Cochrane Review Group, as with CS-COUSIN in dermatology,[22 23] could offer easier access to information about outcomes previously measured in trials, and to Cochrane's international network of consumers, healthcare professionals, and researchers in the field. Additionally, close interaction between COS development and CRGs should support COS implementation. For the dermatology outcome research community, the formal affiliation of COS development groups to Cochrane is

For peer review only - http://bmjopen.bmj.com/site/about/guidelines.xhtml

considered an important advantage. This strategy has led to the affiliation of 15 COS groups to CS-COUSIN since its initiation in 2015.[22 23]

Our study has some limitations. We achieved a 73% response rate from CRG Co-ordinating Editors. Whilst this rate is acceptable, it is conceivable that non-response bias may have arisen as a result of a lack of interest or awareness about COS among editors not responding. A second limitation may have been our assumption that if a COS had been published two or more years before the publication of the systematic review, it would have been available to the reviewers to adopt. This may not have always been true. Previous studies have found that reviews generally,[28] and Cochrane Reviews specifically,[29] take an average of less than 2 years and 2.4 years to be completed however, suggesting this assumption may not be unreasonable.

Cochrane now recommends that reviewers should consider using COS where these exist[18]. Our study identifies some implications for practice when following this guidance. The COMET database of COS (available online at http://www.comet-initiative.org/), is free, searchable, and covers a range of health fields. Using a given COS for a given systematic review involves, at the least, an assessment of the appropriateness of the topic, scope, stakeholder representation, and currency of the COS. We recommend that, when a potentially appropriate COS exists for a given systematic review, the authors should either use it to inform their choice of outcomes or justify their reason for not using it. In addition, authors of Cochrane reviews, especially those that identify outcome inconsistency in included studies, should take the opportunity to move their respective fields forward by

For peer review only - http://bmjopen.bmj.com/site/about/guidelines.xhtml

explicitly noting the need for COS, recommending COS development, and participating in the COS development process.

Our study has some implications for research. The assessment presented here will act as a benchmark for subsequent evaluation of the implementation of the guidance in the Cochrane Handbook that is specific to using COS when choosing outcomes for Cochrane reviews. We note that 66% of the review topics, from 31 CRGs, did not have a relevant COS. Future research should include identification of priority areas for COS development.

The use of COS for a systematic review in turn may influence trialists to use that same COS to ensure that their trials can contribute fully to subsequent reviews and meta-analyses. Given the continued problem of outcome inconsistency in studies documented in Cochrane reviews, and the fact that four CRGs have already established links to COS development groups, this topic could be a potential area for strategic development of Cochrane. Such greater uptake of COS across the healthcare research ecosystem could help improve research and thereby improve health care and health.

Figure 1. Published and Ongoing Core Outcome Sets according to Cochrane Review Group

This list of COS was compiled from the annual update of a review of COS studies conducted by the Core Outcome Measures in Effectiveness Trials (COMET) Initiative and was last updated in 2018.

For peer review only

Figure 2. Cochrane Review Group Outcome Choice Policies. Denominators are (n=45) and (n=35) for 2012 and 2019 respectively.

Figure 3. Cochrane Review Group SoF Table Choice Policies. Denominators are (n=45) and (n=35) for 2012 and 2019 respectively.

Acknowledgements: We thank all the Cochrane Review Group Co-ordinating editors who responded to our questions. We are grateful to the reviewers for their comments which have helped us to improve our manuscript.

Funding: This work was supported by an MRC Hub for Trials Methodology Research Network studentship (grant reference MR/L004933/1-Q30); a National Institute for Health Research (NIHR) Senior Investigator Award (NF-SI_0513-10025); and the MRC Trials Methodology Research Partnership (grant reference MR/S014357/1). The views expressed in this article are those of the authors and not necessarily those of the NIHR, or the Department of Health and Social Care.

Competing Interests: PRW and MC are members of the COMET (Core Outcome Measures in Effectiveness Trials) Management Group. JJK and JS are members of the CS-COUSIN (Cochrane Skin – Core Outcome Set Initiative) methods group. The remaining authors declare no competing interests.

Authors' Contributions: PRW conceived the idea for the manuscript. JJK, MC, JS, and PRW developed the questions for the Cochrane Review Group Co-ordinating Editors. The list of

For peer review only - http://bmjopen.bmj.com/site/about/guidelines.xhtml

the total number of completed and ongoing COS with references for each CRG was produced by SG. The requests to the editors in 2019 were administered by JJK. Their responses were analysed by JJK in collaboration with PRW. The review of Cochrane reviews was designed by IJS and PRW. The data were extracted by RdeAO, KH, JJK, TL, IJS and PRW, confirmed by MC, and analysed by IJS. The assessment of relevance of published COS was undertaken by SG and PRW. PRW, JJK and IJS prepared the initial manuscript. All authors commented on and approved the final manuscript before submission.

Data Sharing Statement: All data are available from the corresponding author on reasonable request.

For peer review only

For peer review only - http://bmjopen.bmj.com/site/about/guidelines.xhtml

References

1. Clarke M, Williamson PR. Core outcome sets and systematic reviews. *Systematic Reviews* 2016;5:11.
2. Kirkham JJ, Dwan KM, Altman DG, et al. The impact of outcome reporting bias in randomised controlled trials on a cohort of systematic reviews. *BMJ* 2010;340:c365.
3. Berglas S, Jutai L, MacKean G, et al. Patients' perspectives can be integrated in health technology assessments: an exploratory analysis of CADTH Common Drug Review. *Res Involv Engagem* 2016;2:21.
4. Glasziou P, Altman DG, Bossuyt P, et al. Reducing waste from incomplete or unusable reports of biomedical research. *The Lancet* 2014;383(9913):267-76.
5. Kirkham JJ, Davis K, Altman DG, et al. Core Outcome Set-STAndards for Development: The COS-STAD recommendations. *PLoS Med* 2017;14(11):e1002447. doi: 10.1371/journal.pmed.1002447
6. Saldanha IJ, Lindsley KB, Money S, et al. Outcome choice and definition in systematic reviews leads to few eligible studies included in meta-analyses: a case study. *BMC Med Res Methodol* 2020;20(1):30. doi: 10.1186/s12874-020-0898-2
7. Saldanha IJ, Lindsley K, Do DV, et al. Comparison of Clinical Trial and Systematic Review Outcomes for the 4 Most Prevalent Eye Diseases. *JAMA Ophthalmol* 2017;135(9):933-40.
8. Saldanha IJ, Li T, Yang C, et al. Clinical trials and systematic reviews addressing similar interventions for the same condition do not consider similar outcomes to be important: A case study in HIV/AIDS. *Journal of Clinical Epidemiology* 2017;84:85-94.
9. Kirkham JJ, Gargon E, Clarke M, et al. Can a core outcome set improve the quality of systematic reviews? – a survey of the Co-ordinating Editors of Cochrane review groups. *Trials* 2013;14:21.
10. COMET Initiative Database Search [Available from: http://www.comet-initiative.org/studies/search accessed cited 23 April 2019.
11. Gargon E, Gurung B, Medley N, et al. Choosing important health outcomes for comparative effectiveness research: a systematic review. *PLoS One* 2014;9(6):e99111.
12. Gorst SL, Gargon E, Clarke M, et al. Choosing Important Health Outcomes for Comparative Effectiveness Research: An Updated Review and User Survey. *PLoS One* 2016;11(1):e0146444.
13. Gorst SL, Gargon E, Clarke M, et al. Choosing Important Health Outcomes for Comparative Effectiveness Research: An Updated Review and Identification of Gaps. *PLoS One* 2016;11(12):e0168403.
14. Davis K, Gorst SL, Harman N, et al. Choosing important health outcomes for comparative effectiveness research: An updated systematic review and involvement of low and middle income countries. *PLoS One* 2018;13(2):e0190695.
15. Gargon E, Gorst SL, Harman NL, et al. Choosing important health outcomes for comparative effectiveness research: 4th annual update to a systematic review of core outcome sets for research. *PLoS One* 2018;13(12):e0209869.
16. Gargon E, Gorst SL, Williamson PR. Choosing important health outcomes for comparative effectiveness research: 5th annual update to a systematic review of core outcome sets for research. *PLoS One* 2019;14(12):e0225980. doi: 10.1371/journal.pone.0225980
17. Kirkham JJ, Davis K, Altman DG, et al. Core Outcome Set-STAndards for Development: The COS-STAD recommendations. *PLoS Med* 2017;14(11):e1002447.
18. Kirkham JJ, Gorst S, Altman DG, et al. Core Outcome Set-STAndards for Reporting: The COS-STAR Statement. *PLoS Med* 2016;13(10):e1002148.
19. Kirkham JJ, Gorst SL, Altman DG, et al. Core Outcome Set-STAndardised Protocol Items: The COS-STAP Statement. *Trials* 2019;20:116.

For peer review only - http://bmjopen.bmj.com/site/about/guidelines.xhtml

20. Williamson PR, Altman DG, Bagley H, et al. The COMET Handbook: version 1.0. *Trials* 2017;18(Suppl 3):280.
21. Higgins JPT, Thomas J, Chandler J, et al., editors. *Cochrane Handbook for Systematic Reviews of Interventions. 2nd Edition*. Chichester (UK): John Wiley & Sons, 2019.
22. Cochrane Skin: Core Outcome Set Initiative (CS-COUSIN) [Available from: http://cs-cousin.org/ accessed cited 23 April 2019.
23. Prinsen CAC, Spuls PI, Kottner J, et al. Navigating the landscape of core outcome set development in dermatology. *J Am Acad Dermatol* 2019;81(1):297-305.
24. Hughes KL, Kirkham JJ, Clarke M, et al. Assessing the impact of a research funder's recommendation to consider core outcome sets. *PLoS One* 2019;14(9):e0222418. doi: 10.1371/journal.pone.0222418
25. OMERACT Initiative [Available from: https://omeract.org/ accessed cited 23 April 2019.
26. SONG Initiative [Available from: https://songinitiative.org/ accessed cited 23 April 2019.
27. CROWN Initiative [Available from: http://www.crown-initiative.org/ accessed cited 23 April 2019.
28. Borah R, Brown AW, Capers PL, et al. Analysis of the time and workers needed to conduct systematic reviews of medical interventions using data from the PROSPERO registry. *BMJ Open* 2017;7(2):e012545. doi: 10.1136/bmjopen-2016-012545
29. Tricco AC, Brehaut J, Chen MH, et al. Following 411 Cochrane protocols to completion: a retrospective cohort study. *PLoS One* 2008;3(11):e3684. doi: 10.1371/journal.pone.0003684

For peer review only

For peer review only - http://bmjopen.bmj.com/site/about/guidelines.xhtml

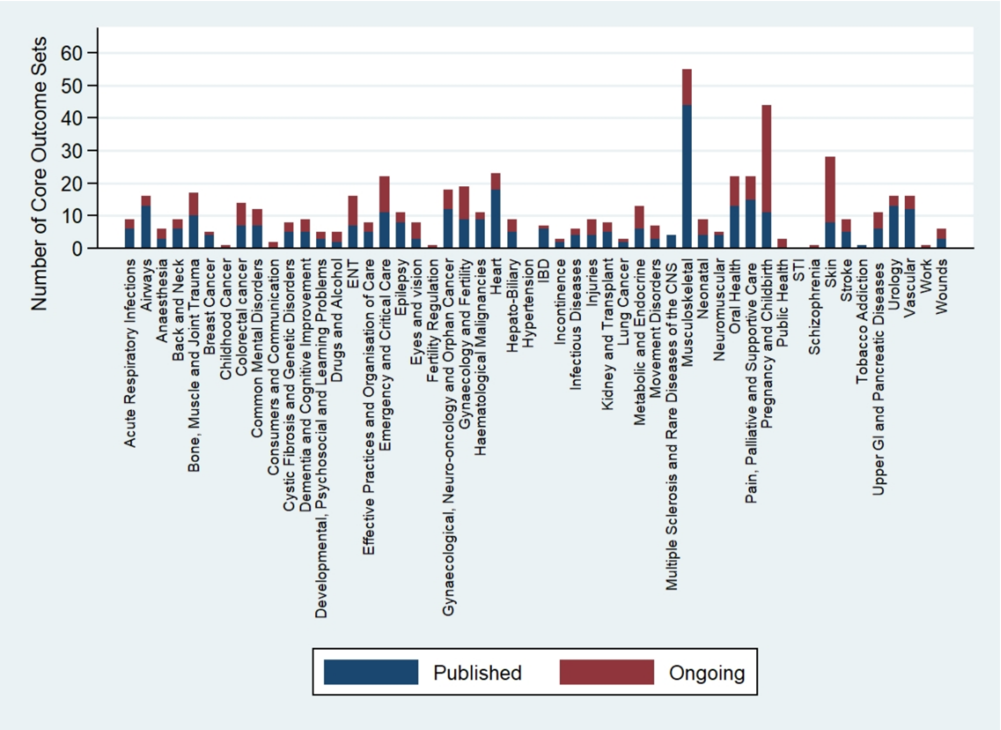

Figure 1. Published and Ongoing Core Outcome Sets according to Cochrane Review Group

108x78mm (300 x 300 DPI)

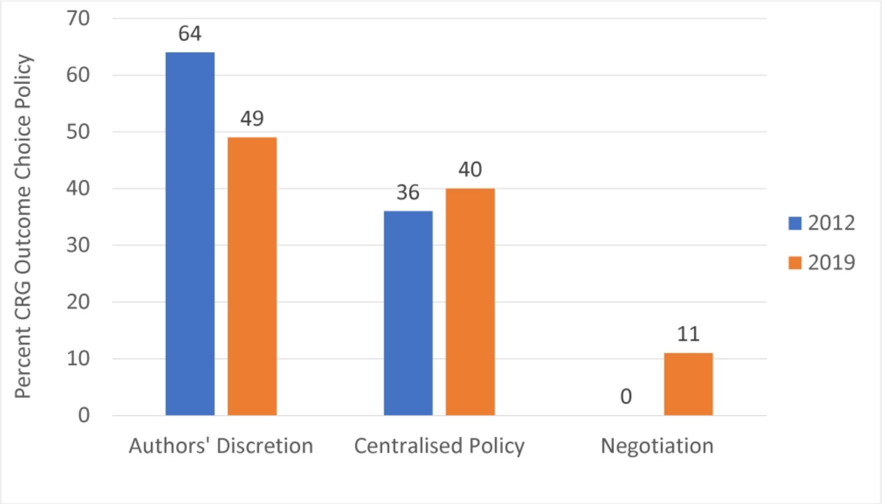

Figure 2. Cochrane Review Group Outcome Choice Policies. Denominators are (n=45) and (n=35) for 2012 and 2019 respectively.

297x194mm (300 x 300 DPI)

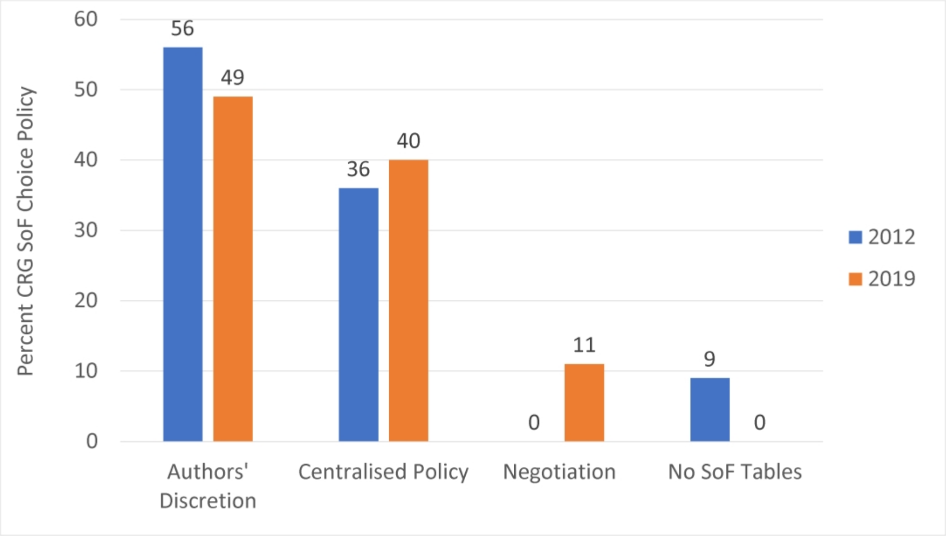

Figure 3. Cochrane Review Group SoF Table Choice Policies. Denominators are (n=45) and (n=35) for 2012 and 2019 respectively.

297x193mm (300 x 300 DPI)

Assessing the relevance and uptake of core outcome sets (an agreed minimum collection of outcomes to measure in research studies) in Cochrane systematic reviews: a review

Journal:	*BMJ Open*
Manuscript ID	bmjopen-2019-036562.R2
Article Type:	Original research
Date Submitted by the Author:	29-Jul-2020
Complete List of Authors:	Williamson, Paula; University of Liverpool,<br>de Ávila Oliveira, Ricardo; Universidade Federal de Uberlândia<br>Clarke, Mike; Queen's University Belfast, Northern Ireland Methodology Hub<br>Gorst, Sarah L.; University of Liverpool<br>Hughes, Karen; University of Liverpool<br>Kirkham, Jamie J.; University of Liverpool, Biostatistics<br>Li, Tianjing; University of Colorado<br>Saldanha, Ian; Brown University<br>Schmitt, Jochen; Technische Universität Dresden, Department of Dermatology
**Primary Subject Heading**:	Health services research
Secondary Subject Heading:	Research methods
Keywords:	STATISTICS & RESEARCH METHODS, Clinical trials < THERAPEUTICS, GENERAL MEDICINE (see Internal Medicine)

SCHOLARONE™
Manuscripts

I, the Submitting Author has the right to grant and does grant on behalf of all authors of the Work (as defined in the below author licence), an exclusive licence and/or a non-exclusive licence for contributions from authors who are: i) UK Crown employees; ii) where BMJ has agreed a CC-BY licence shall apply, and/or iii) in accordance with the terms applicable for US Federal Government officers or employees acting as part of their official duties; on a worldwide, perpetual, irrevocable, royalty-free basis to BMJ Publishing Group Ltd ("BMJ") its licensees and where the relevant Journal is co-owned by BMJ to the co-owners of the Journal, to publish the Work in this journal and any other BMJ products and to exploit all rights, as set out in our licence.

The Submitting Author accepts and understands that any supply made under these terms is made by BMJ to the Submitting Author unless you are acting as an employee on behalf of your employer or a postgraduate student of an affiliated institution which is paying any applicable article publishing charge ("APC") for Open Access articles. Where the Submitting Author wishes to make the Work available on an Open Access basis (and intends to pay the relevant APC), the terms of reuse of such Open Access shall be governed by a Creative Commons licence – details of these licences and which Creative Commons licence will apply to this Work are set out in our licence referred to above.

Other than as permitted in any relevant BMJ Author's Self Archiving Policies, I confirm this Work has not been accepted for publication elsewhere, is not being considered for publication elsewhere and does not duplicate material already published. I confirm all authors consent to publication of this Work and authorise the granting of this licence.

For peer review only

Assessing the relevance and uptake of core outcome sets (an agreed minimum collection of outcomes to measure in research studies) in Cochrane systematic reviews: a review

Paula R Williamson[a*]

Ricardo de Ávila Oliveira[b]

Mike Clarke[c]

Sarah L Gorst[a]

Karen Hughes[a]

Jamie J Kirkham[d]

Tianjing Li[e]

Ian J Saldanha[f]

Jochen Schmitt[g]

[a] MRC North West Hub for Trials Methodology Research, University of Liverpool and member of Liverpool Health Partners, Liverpool, United Kingdom

[b] DECIR Faculdade de Medicina, Universidade Federal de Uberlândia, Uberlândia, Brazil

[c] Northern Ireland Methodology Hub, Centre for Public Health, Queen's University Belfast, Belfast, United Kingdom

[d] Centre for Biostatistics, Manchester Academic Health Science Centre, University of Manchester, Manchester, United Kingdom

[e] Department of Ophthalmology, University of Colorado Denver, Aurora, Colorado, USA

[f] Center for Evidence Synthesis in Health, Brown University School of Public Health, Providence, Rhode Island, USA

For peer review only - http://bmjopen.bmj.com/site/about/guidelines.xhtml

[g] Center for Evidence-based Healthcare, Medizinische Fakultät, Technische Univesität Dresden, Dresden, Germany

[*]**Corresponding Author:**

Professor Paula Williamson

Department of Biostatistics

University of Liverpool

Block F Waterhouse Building, 1-5 Brownlow Street, Liverpool, L69 3GL, United Kingdom

Email: prw@liv.ac.uk

Tel: +44 (0) 151 794 9758

Word Count: 3320

For peer review only

For peer review only - http://bmjopen.bmj.com/site/about/guidelines.xhtml

ABSTRACT

Objectives: A core outcome set (COS) is an agreed standardised minimum collection of outcomes that should be measured and reported in research in a specific area of health. Cochrane systematic reviews ("reviews") are rigorous reviews on health-related topics conducted under the auspices of Cochrane. This study examines the use of existing COS to inform the choice of outcomes in Cochrane systematic reviews ("reviews") and investigates the views of the Co-ordinating Editors of Cochrane Review Groups on this topic.

Methods: A cohort of 100 recently published or updated Cochrane reviews were assessed for reference to a COS being used to inform the choice of outcomes for the review. Existing COS, published two or more years before the review publication, were then identified to assess how often a reviewer could have used a relevant COS if it was available. We asked 52 Cochrane Review Group (CRG) Co-ordinating Editors about their involvement in COS development, how outcomes are selected for reviews in their CRG, and their views of the advantages and challenges surrounding the standardisation of outcomes within their CRG.

Results: In the cohort of reviews from 2019, 40% (40/100) of reviewers noted problems due to outcome inconsistency across the included studies. In 7% (7/100) of reviews, a COS was referenced in relation to the choice of outcomes for the review. Relevant existing COS could be considered for a review update in 35% of the others (33/93). Most editors who responded (31/36, 86%) thought that COS should definitely or possibly be used to inform the choice of outcomes in a review.

For peer review only - http://bmjopen.bmj.com/site/about/guidelines.xhtml

Conclusions: Systematic reviewers are continuing to note outcome heterogeneity but are starting to use COS to inform their reviews. There is potential for greater uptake of COS in Cochrane reviews.

Keywords: Cochrane Review Group; Co-ordinating Editor; core outcome set; summary of findings; systematic reviews

STRENGTHS AND LIMITATIONS OF THIS STUDY

- A sample of 100 Cochrane systematic reviews that were published for the first time or updated in 2019 were assessed.
- Two investigators independently assessed the relevance of a core outcome set (COS) to each systematic review.
- An assumption was made that if a COS had been published 2 or more years before the publication of the systematic review, it would have been available to the reviewers to adopt.
- Co-ordinating Editors for all Cochrane Review Groups were asked for their views on the relevance of COS to systematic reviews.
- The research was undertaken before the release of version 6 of the Cochrane Handbook for Systematic Reviews of Interventions, which recommends that Cochrane reviewers should consider using a COS.

For peer review only - http://bmjopen.bmj.com/site/about/guidelines.xhtml

BACKGROUND

Systematic reviews ("reviews") of the effects of healthcare interventions summarise the available evidence and are used to inform decision-making. Inconsistencies in the outcomes that are measured and reported across studies included in reviews can mean that it is not possible for all studies to be included in a meta-analysis however, a form of research waste. In addition, the selective reporting of outcomes by some included studies based on the direction or magnitude of the results, a phenomenon known as outcome reporting bias, can affect the robustness of the conclusions of a review.[1,2]

Furthermore, there is increasing awareness that differences exist between the outcomes measured in clinical trials and outcomes that patients consider important. For example, only 50% of the outcomes that patients said matter to them were captured in the clinical trials reviewed by the Canadian Agency for Drugs and Technologies in Health (CADTH) Common Drug Review.[3] Such problems also contribute to research waste.[4]

One solution to these problems with the choice of outcomes is for clinical trialists in a particular topic area to measure and report, at a minimum, a core outcome set (COS), which would then be used in reviews addressing that same topic area. A COS is an agreed standardised collection of outcomes that should be measured and reported in a specific area of health. It does not preclude the inclusion of additional outcomes, but represents the minimum for all research studies in the topic area. The scope of a COS refers to the specific area of health or healthcare of interest to which the COS is to be applied, defined in terms of the health condition, population, and healthcare interventions covered. Thus, within a particular topic area there may be multiple COS, depending on whether a COS is developed

For peer review only - http://bmjopen.bmj.com/site/about/guidelines.xhtml

for different populations, for example adults versus children, or localised versus advanced disease. A COS may be developed to apply to any intervention for that condition, or it may be for specific types, for example surgery.

Minimum standards have been established for developing COS.[5] According to these standards, as a minimum, the stakeholders who should be involved in developing a COS should be those with lived experience of the condition, health professionals caring for those with lived experience, and those undertaking research in the condition.[5]

The benefits of widespread adoption of a COS are to increase outcome consistency across trials, resulting in a major reduction in selective reporting and maximising the potential for a trial to contribute to meta-analyses of these key outcomes.[6] Of crucial importance is that, given the expectations of relevant stakeholder involvement, the use of a COS should mean trialists are much more likely to measure appropriate outcomes that are relevant to patients.

An additional issue in the choice of outcomes for a systematic review relates to clinical trialists and systematic reviewers sometimes being interested in different types of outcomes.[7 8] Clarke and Williamson have argued for greater involvement of systematic reviewers in the development and implementation of COS,[1] suggesting this might help with planning of reviews and ensuring that reviews identify data for outcomes of interest.

For peer review only - http://bmjopen.bmj.com/site/about/guidelines.xhtml

Cochrane reviews are rigorous systematic reviews conducted under the auspices of Cochrane, a global and independent volunteer organisation with the primary goal of conducting evidence syntheses in health. Cochrane is organised operationally into Cochrane Review Groups (CRGs), comprised of an editorial team, information specialists, and authors responsible for reviews within distinct clinical and public health areas. For specific reviews, decisions need to be made regarding outcomes that will be included in the review as well as the subset of key outcomes that will be prioritised for inclusion in the Summary of Findings (SoF) tables. SoF tables are intended to provide consumers of the review with the key findings of the review.

Each CRG is led by a Co-ordinating Editor who oversees the production and maintenance of Cochrane reviews. In a 2012 survey of CRG Co-ordinating Editors (hereafter “editors”), the majority (33/45, 73%) thought that COS should routinely be used in the SoF tables.[9] At that time, a third of the editors (14/45, 31%) reported personal involvement in the development of a COS.

The awareness and development of COS have substantially increased over recent years, with more than 100 new COS published between 2012 and 2019, and at least 200 more in development.[10-16] A series of guidelines have been published to improve the design and reporting of COS development.[17-20] The relevance of COS to defining review questions and planning the review, as well as the inclusion of patient-centred outcomes within COS, is acknowledged in the 2019 version of the Cochrane Handbook for Systematic Reviews of Interventions, which states, “where available, established sets of core outcomes should be used”.[21] One notable link between COS development and Cochrane is the work of the

For peer review only - http://bmjopen.bmj.com/site/about/guidelines.xhtml

For peer review only

Cochrane Skin Group on the Cochrane Skin Core Outcome Set Initiative (CS-COUSIN).[22 23] The mission of this group is to standardise outcomes in dermatology clinical trials in order to make trial evidence more comparable and, thereby, to strengthen the quality, interpretability, and ability of systematic reviews to facilitate evidence-based decision-making in dermatology.

In this paper, we examine current practices of Cochrane systematic reviewers in relation to the use of COS in choosing outcomes, and the current views of Cochrane Review Group editors regarding the adoption of COS in their CRGs.

METHODS

Assessment of Cochrane reviews in relation to COS

Although a formal protocol was not developed for this study, the following approach was agreed amongst the research team in advance. We examined the first 100 new or updated Cochrane intervention reviews published in 2019 (dated 1st January to 8th March). We chose to restrict to reviews published in 2019 in order to determine contemporary practices regarding outcome choice in Cochrane reviews. The sample size was a pragmatic choice, to give a reasonable number of reviews that we could assess in a reasonable period of time.

We examined whether, in the completed version of the review, the review authors: (i) mentioned using a COS to choose outcomes for the review, even if all the outcomes in the COS may not have been used; (ii) identified any problems with outcome inconsistency across the studies included in the review; and (iii) noted the need for development of a COS. We extracted information that may have been reported in any section of the completed

For peer review only - http://bmjopen.bmj.com/site/about/guidelines.xhtml

review. One investigator (from among IJS, JJK, KH, PRW, RdeAO, and TL) extracted information from each review and one investigator (from among MC and IJS) verified the extracted information.

For each review, we searched the COMET database (a regularly-updated online repository of COS studies) to establish whether a COS with a relevant scope (even if not an exact match) had been published by 31 December 2016, such that it might have been available to inform outcome choice at the planning stage for reviews that were published more than 2 years later. For reviews where no such COS was found, the COMET database was also searched for COS published since 1 January 2017, to determine whether a relevant COS may be available for consideration when the Cochrane review is updated. Two investigators (SLG and PRW) assessed the potential relevance of identified COS independently and then compared their assessments, following an approach used previously (data available on request).[24]

Co-ordinating Editors of Cochrane Review Groups

Consistent with the approach used in our 2012 survey,[7] we emailed the editors of all 52 CRGs (as of 2019) requesting them to provide information on their involvement with COS development and their CRG policy with regards to outcome selection for reviews and prioritisation of outcomes for associated SoF tables. They were presented with a breakdown of the total number of completed and ongoing COS for research, with references, that were within the scope of their CRG (Figure 1 summarises the number of COS by topic area).

Editors were also asked about their opinions on the standardisation of outcomes within their CRG, including what they thought were up to three main advantages and three main

For peer review only - http://bmjopen.bmj.com/site/about/guidelines.xhtml

challenges of standardising outcomes across all reviews in a particular condition covered by their CRG. They were provided with the responses from the 2012 survey for their CRG and were asked to indicate whether those earlier responses remained the same or to identify any changes of opinion.

Expanding on our 2012 survey, we requested them to provide details of any examples where a COS had been considered to guide the choice of outcomes to include in a Cochrane review or a SoF table. Second, we described the aims of the CS-COUSIN initiative and asked editors for their views on the relevance and feasibility of establishing links between their CRG and an equivalent COS group.

Responses were collected between 26th February and April 30th, 2019. Non-responders were contacted every 3 weeks during this period with reminders to participate.

Analysis

The data from the assessment of Cochrane reviews were analysed descriptively. The lists of advantages and challenges of COS suggested by editors was independently reviewed by two authors (JJK and PRW) and coded using the categories identified in the 2012 survey, allowing new categories to be added. Discrepancies in categorisation were resolved through discussion.

Patient and public involvement

Patients or the public were not involved in the design, conduct, or reporting of this research.

For peer review only - http://bmjopen.bmj.com/site/about/guidelines.xhtml

RESULTS

Assessment of Cochrane reviews in relation to use of COS to inform outcome choice

The reviews assessed addressed a range of health conditions (Table 1). Forty-one of the 52 current CRGs (79%) were represented. Forty reviews (40%) identified problems of inconsistency in outcomes in included studies; in five of these, the authors also explicitly noted the need for development of a COS.

Table 1. Health conditions addressed in the 100 Cochrane Reviews assessed in relation to use of COS

ICD-11 Code	Disease area	N (%)
12	Respiratory system	11 (11%)
16	Genitourinary system	11 (11%)
2	Neoplasms	9 (9%)
11	Circulatory system	8 (8%)
1	Infectious or parasitic diseases	5 (5%)
8	Nervous system	5 (5%)
13	Digestive system	4 (4%)
15	Musculoskeletal system	4 (4%)
9	Visual system	4 (5%)
14	Skin	3 (3%)
24	Factors influencing health status or contact with health services	3 (3%)
4	Immune system	2 (2%)
5	Endocrine, nutritional, or metabolic diseases	2 (2%)
6	Mental, behavioural, or neurodevelopmental	2 (2%)
7	Sleep-wake disorders	2 (2%)
17	Conditions related to sexual health	2 (2%)
3	Blood or blood-forming organs	1 (1%)
10	Ear or mastoid process	1 (1%)
19	Certain conditions originating in the perinatal period	1 (1%)
21	Symptoms, signs or clinical findings, not elsewhere classified	1 (1%)
22	Injury, poisoning, or certain other consequences of external causes	1 (1%)
23	External causes of morbidity or mortality	1 (1%)
No specific ICD-11 code	Miscellaneous	17 (17%)

For peer review only - http://bmjopen.bmj.com/site/about/guidelines.xhtml

For peer review only

Seven of 100 (7%) reviews mentioned (or cited) a COS in relation to choosing outcomes for the review. A further three reviews, while not mentioning a COS in relation to choosing outcomes for the review and not specifically recommending the use of a COS, made observations about existing COS. One review noted that several patient-important outcomes identified in an existing COS were absent from most of the review's included studies. One review acknowledged an existing COS, noting that the trials in the review continued to report inconsistent outcomes, and noted that further work was needed on how the outcome domains in the COS should be measured to facilitate future reviews. The third review recommended that researchers should adopt the definition of a particular outcome domain of interest to the review as provided in an existing COS.

For the 90 reviews not referring to a COS, a relevant COS was found to have been published in 2016 or earlier in 24. Thus, of 93 reviews not referencing a COS in relation to choosing outcomes for the review, in 27 (29%) cases, this could have been possible.

In summary, of the 100 reviews, a COS from 2016 or earlier was available for 34 (34%). Of those 34 reviews, a COS was used in 7 (21%). In a further 6 reviews, a relevant COS was published in 2017 or later. Thus, a relevant COS could be considered during the updating of at least 35% (33/93) of the reviews that have not already used a COS.

For peer review only - http://bmjopen.bmj.com/site/about/guidelines.xhtml

Editors' views

Thirty-eight (73%) of the 52 CRG editors responded to our emails, although some did not provide a response to all questions. Two-thirds of the editors (23/35, 66%) had been involved in the development of a COS, eight editors prior to the 2012 survey, and 15 since then.

As of 23rd April 2019, the COMET database contained 323 published and 242 ongoing COS across all CRGs, ranging from 0 (Hypertension and Sexually Transmitted Infections) to 54 (Musculoskeletal) (Figure 1). The total number of published and ongoing COS was slightly higher for CRGs whose editors responded in 2019 (median 9, interquartile range [IQR] 5 to 16.5) compared with not (median 7, IQR 5 to 9).

Of 22 editors who responded in both 2012 and 2019, 64% noted in 2012 that COS should be used in SoF; this percentage rose to 91% in 2019.

Cochrane Review Group Outcome Policies

Figures 2 and 3 show the policies of CRGs for outcome selection for reviews (Figure 2) and for SoF tables (Figure 3); both show a reduction in a policy of authors' discretion since 2012, to 49% in 2019. Currently, 40% (14/35) of CRGs have a centralised policy for both review and SoF table outcome choice. Four editors (11%) indicated that outcome choice was now a negotiation between authors and the editorial team, with input from peer reviewers.

In 2019, half of the editors (18/36; 50%) thought that COS should definitely be considered in the process of choosing outcomes for a SoF, whilst a further 13 (36%) editors thought that

For peer review only - http://bmjopen.bmj.com/site/about/guidelines.xhtml

For peer review only

COS could possibly be used in some circumstances if relevant to the specific review and if the COS was developed using a 'quality' consensus approach. Two (6%) editors thought that COS should not be used in the SoF tables because they can lead to the inclusion of unnecessary outcomes. One editor did not think that the inclusion of COS in SoF tables was relevant, because they did not think COS were used in their topic area. Two editors were unsure because they were aware that many COS contain more than the maximum of seven outcomes that Cochrane allows for SoF tables.

Advantages and Challenges of Standardising Outcomes in Cochrane Reviews

For both the advantages and challenges associated with standardising outcomes across reviews (Table 2), the three most commonly listed were the same as in the 2012 survey. Just under half of the editors consider an advantage of using a COS to be that the outcomes are likely to be more appropriate.

Table 2. Views of Cochrane Editors on standardising outcomes across all reviews for a particular condition

**Advantages**	**2012 n/40 (%*)**	**2019 n/32 (%*)**
Advantage for a systematic review/meta-analysis	39 (98)	27 (84)
Improves interpretation/guidance	19 (48)	7 (22)
Outcome likely to be more appropriate	16 (40)	10 (45)
Advantage for the design of a new study	13 (33)	5 (16)
Improves something about the outcome itself (e.g. simplifies the reporting)	6 (15)	0 (0)
Reduces outcome reporting bias	6 (15)	1 (3)
Reduces resource requirement (e.g. time to review)	1 (3)	1 (3)
**Challenges**	**2012 n/42 (%*)**	**2019 n/36 (%*)**
Development of a COS	23 (55)	8 (22)
Something about scope	21 (50)	28 (78)
How to persuade authors/trialists/industry to implement	20 (48)	14 (39)
'How' to measure once the 'what' has been decided	11 (26)	3 (8)

For peer review only - http://bmjopen.bmj.com/site/about/guidelines.xhtml

For peer review only

Important outcomes not currently being measured	2 (5)	1 (3)
Resource to develop	2 (5)	3 (8)
Updating process	2 (5)	2 (6)
Conflict of interest	1 (1)	0 (0)
Limits authors	0 (2)	3 (8)

*Percentages represent the number of Cochrane Review Group Co-ordinating Editors who mentioned each advantage/challenge

One notable difference from 2012 was a shift from the most frequently-listed challenge being COS development (55% in 2012, falling to third place on 22% in 2019) to deciding when a COS should be applied (in relation to the scope of the review and the scope of the COS) in 2019 (78%). One new challenge identified in 2019 by three editors (8%) was the perception that the use of a COS may limit review authors due to a lack of flexibility in outcome choice.

Links between CRG, COS developers, and guidance

Eight editors identified cases where the outcome choice in at least one review in their group had been informed by a COS. The majority (22/26, 85%) of editors thought that links between their CRG and COS developers in their field would be a good idea in principle, whilst four could see no benefit. In addition to the Cochrane Skin Group, three CRGs had already established such links: the Cochrane Musculoskeletal Group with the Outcome Measures in Rheumatology (OMERACT) Initiative,[25] the Cochrane Kidney and Transplant Group with the Standardising Outcomes in Nephrology (SONG) Initiative,[26] and the Cochrane Pregnancy and Childbirth Group with the Core Outcomes in Women's and Newborn Health (CROWN) Initiative.[27]

For peer review only - http://bmjopen.bmj.com/site/about/guidelines.xhtml

The major perceived barriers to establishing links between CRGs and COS developers were related to lack of resources, mainly lack of time (mentioned nine times) and funding to co-ordinate (mentioned five times). Conflicts of interest, the potential for poor communication between members, and lack of knowledge on how to manage such links were also quoted as potential barriers to implementation.

CONCLUSIONS

Our study has demonstrated that Cochrane systematic reviewers note widespread outcome inconsistency across studies. We found that almost all CRG editors think that COS should definitely or possibly be used in SoF tables, and that reviewers are beginning to use COS to inform their choice of outcomes.

We have documented an increased level of involvement of CRG editors in COS development studies since 2012. The COS-STAD (Core Outcome Set-STAndards for Development) guidance identifies three stakeholder groups as the minimum for input into the development of a COS: patients or their representatives, healthcare professionals, and those who will use the COS in research.[17] Consensus was not quite achieved that a fourth group, those who will use the research that should use the COS (e.g., systematic reviewers, guideline developers, policy makers, regulatory agencies), should always be involved. COS-STAD participants may have wanted to avoid setting a minimum standard when there was limited experience of engaging with these particular stakeholder groups. If the involvement of Cochrane reviewers in COS development studies continues to increase, it would seem appropriate to review the standard in due course.

For peer review only - http://bmjopen.bmj.com/site/about/guidelines.xhtml

The majority of editors were in favour of cooperation between their CRG and COS developers in their field, however the most frequently mentioned potential barriers, such as lack of time, resources, and funding, will need to be addressed to achieve this. The affiliation of a COS development initiative to a Cochrane Review Group, as with CS-COUSIN in dermatology,[22 23] could offer easier access to information about outcomes previously measured in trials, and to Cochrane's international network of consumers, healthcare professionals, and researchers in the field. Additionally, close interaction between COS development and CRGs should support COS implementation. For the dermatology outcome research community, the formal affiliation of COS development groups to Cochrane is considered an important advantage. This strategy has led to the affiliation of 15 COS groups to CS-COUSIN since its initiation in 2015.[22 23]

Our study has some limitations. No formal protocol was developed for this study, however the research team had agreed the methodological approach prior to commencing the study. Second, we achieved a 73% response rate from CRG Co-ordinating Editors. Whilst this rate is acceptable, it is conceivable that non-response bias may have arisen as a result of a lack of interest or awareness about COS among editors not responding. A third limitation may have been our assumption that if a COS had been published 2 or more years before the publication of the systematic review, it would have been available to the reviewers to adopt. This may not have always been true. Previous studies have found that reviews generally,[28] and Cochrane Reviews specifically,[29] take an average of less than 2 years and 2.4 years to be completed, respectively. This suggests that our assumption regarding availability of the COS to the systematic reviewers may not be unreasonable.

For peer review only - http://bmjopen.bmj.com/site/about/guidelines.xhtml

Cochrane now recommends that reviewers should consider using COS where these exist.[18] Our study identifies some implications for practice when following this guidance. The COMET database of COS (available online at http://www.comet-initiative.org/), is free, searchable, and covers a range of health fields. Using a given COS for a given systematic review involves, at the least, an assessment of the appropriateness of the topic, scope, stakeholder representation, and currency of the COS. We recommend that, when a potentially appropriate COS exists for a given systematic review, the authors should either use it to inform their choice of outcomes or justify their reason for not using it. In addition, authors of Cochrane reviews, especially those that identify outcome inconsistency in included studies, should take the opportunity to move their respective fields forward by explicitly noting the need for COS, recommending COS development, and participating in the COS development process.

Our study has some implications for research. The assessment presented here will act as a benchmark for subsequent evaluation of the implementation of the guidance in the Cochrane Handbook that is specific to using COS when choosing outcomes for Cochrane reviews. We note that 66% of the review topics, from 31 CRGs, did not have a relevant COS. Future research should include identification of priority areas for COS development.

The use of COS for a systematic review in turn may influence clinical trialists to use that same COS to ensure that their trials can contribute fully to subsequent reviews and meta-analyses. Given the continued problem of outcome inconsistency in studies documented in Cochrane reviews, and the fact that four CRGs have already established links to COS development groups, this topic could be a potential area for strategic development of

For peer review only

For peer review only - http://bmjopen.bmj.com/site/about/guidelines.xhtml

Cochrane. Such greater uptake of COS across the healthcare research ecosystem could help improve research and thereby improve healthcare and health.

Figure 1. Published and Ongoing Core Outcome Sets according to Cochrane Review Group

This list of COS was compiled from the annual update of a review of COS studies conducted by the Core Outcome Measures in Effectiveness Trials (COMET) Initiative and was last updated in 2018.

Figure 2. Cochrane Review Group Outcome Choice Policies. Denominators are (n=45) and (n=35) for 2012 and 2019 respectively.

Figure 3. Cochrane Review Group SoF Table Choice Policies. Denominators are (n=45) and (n=35) for 2012 and 2019 respectively.

Acknowledgements: We thank all the Cochrane Review Group Co-ordinating editors who responded to our questions. We are grateful to the reviewers for their comments which have helped us to improve our manuscript.

Funding: This work was supported by an MRC Hub for Trials Methodology Research Network studentship (grant reference MR/L004933/1-Q30); a National Institute for Health Research (NIHR) Senior Investigator Award (NF-SI_0513-10025); and the MRC Trials Methodology Research Partnership (grant reference MR/S014357/1). The views expressed

For peer review only

in this article are those of the authors and not necessarily those of the NIHR, or the Department of Health and Social Care.

Competing Interests: PRW and MC are members of the COMET (Core Outcome Measures in Effectiveness Trials) Management Group. JJK and JS are members of the CS-COUSIN (Cochrane Skin – Core Outcome Set Initiative) methods group. The remaining authors declare no competing interests.

Authors' Contributions: PRW conceived the idea for the manuscript. JJK, MC, JS, and PRW developed the questions for the Cochrane Review Group Co-ordinating Editors. The list of the total number of completed and ongoing COS with references for each CRG was produced by SG. The requests to the editors in 2019 were administered by JJK. Their responses were analysed by JJK in collaboration with PRW. The review of Cochrane reviews was designed by IJS and PRW. The data were extracted by RdeAO, KH, JJK, TL, IJS and PRW, confirmed by MC, and analysed by IJS. The assessment of relevance of published COS was undertaken by SG and PRW. PRW, JJK and IJS prepared the initial manuscript. All authors commented on and approved the final manuscript before submission.

Data Sharing Statement: All data are available from the corresponding author on reasonable request.

For peer review only - http://bmjopen.bmj.com/site/about/guidelines.xhtml

References

1. Clarke M, Williamson PR. Core outcome sets and systematic reviews. *Systematic Reviews* 2016;5:11.
2. Kirkham JJ, Dwan KM, Altman DG, et al. The impact of outcome reporting bias in randomised controlled trials on a cohort of systematic reviews. *BMJ* 2010;340:c365.
3. Berglas S, Jutai L, MacKean G, et al. Patients' perspectives can be integrated in health technology assessments: an exploratory analysis of CADTH Common Drug Review. *Res Involv Engagem* 2016;2:21.
4. Glasziou P, Altman DG, Bossuyt P, et al. Reducing waste from incomplete or unusable reports of biomedical research. *The Lancet* 2014;383(9913):267-76.
5. Kirkham JJ, Davis K, Altman DG, et al. Core Outcome Set-STAndards for Development: The COS-STAD recommendations. *PLoS Med* 2017;14(11):e1002447. doi: 10.1371/journal.pmed.1002447
6. Saldanha IJ, Lindsley KB, Money S, et al. Outcome choice and definition in systematic reviews leads to few eligible studies included in meta-analyses: a case study. *BMC Med Res Methodol* 2020;20(1):30. doi: 10.1186/s12874-020-0898-2
7. Saldanha IJ, Lindsley K, Do DV, et al. Comparison of Clinical Trial and Systematic Review Outcomes for the 4 Most Prevalent Eye Diseases. *JAMA Ophthalmol* 2017;135(9):933-40.
8. Saldanha IJ, Li T, Yang C, et al. Clinical trials and systematic reviews addressing similar interventions for the same condition do not consider similar outcomes to be important: A case study in HIV/AIDS. *Journal of Clinical Epidemiology* 2017;84:85-94.
9. Kirkham JJ, Gargon E, Clarke M, et al. Can a core outcome set improve the quality of systematic reviews? – a survey of the Co-ordinating Editors of Cochrane review groups. *Trials* 2013;14:21.
10. COMET Initiative Database Search [Available from: http://www.comet-initiative.org/studies/search accessed cited 23 April 2019.
11. Gargon E, Gurung B, Medley N, et al. Choosing important health outcomes for comparative effectiveness research: a systematic review. *PLoS One* 2014;9(6):e99111.
12. Gorst SL, Gargon E, Clarke M, et al. Choosing Important Health Outcomes for Comparative Effectiveness Research: An Updated Review and User Survey. *PLoS One* 2016;11(1):e0146444.
13. Gorst SL, Gargon E, Clarke M, et al. Choosing Important Health Outcomes for Comparative Effectiveness Research: An Updated Review and Identification of Gaps. *PLoS One* 2016;11(12):e0168403.
14. Davis K, Gorst SL, Harman N, et al. Choosing important health outcomes for comparative effectiveness research: An updated systematic review and involvement of low and middle income countries. *PLoS One* 2018;13(2):e0190695.
15. Gargon E, Gorst SL, Harman NL, et al. Choosing important health outcomes for comparative effectiveness research: 4th annual update to a systematic review of core outcome sets for research. *PLoS One* 2018;13(12):e0209869.
16. Gargon E, Gorst SL, Williamson PR. Choosing important health outcomes for comparative effectiveness research: 5th annual update to a systematic review of core outcome sets for research. *PLoS One* 2019;14(12):e0225980. doi: 10.1371/journal.pone.0225980
17. Kirkham JJ, Davis K, Altman DG, et al. Core Outcome Set-STAndards for Development: The COS-STAD recommendations. *PLoS Med* 2017;14(11):e1002447.
18. Kirkham JJ, Gorst S, Altman DG, et al. Core Outcome Set-STAndards for Reporting: The COS-STAR Statement. *PLoS Med* 2016;13(10):e1002148.
19. Kirkham JJ, Gorst SL, Altman DG, et al. Core Outcome Set-STAndardised Protocol Items: The COS-STAP Statement. *Trials* 2019;20:116.

For peer review only - http://bmjopen.bmj.com/site/about/guidelines.xhtml

20. Williamson PR, Altman DG, Bagley H, et al. The COMET Handbook: version 1.0. *Trials* 2017;18(Suppl 3):280.
21. Higgins JPT, Thomas J, Chandler J, et al., editors. *Cochrane Handbook for Systematic Reviews of Interventions. 2nd Edition*. Chichester (UK): John Wiley & Sons, 2019.
22. Cochrane Skin: Core Outcome Set Initiative (CS-COUSIN) [Available from: http://cs-cousin.org/ accessed cited 23 April 2019.
23. Prinsen CAC, Spuls PI, Kottner J, et al. Navigating the landscape of core outcome set development in dermatology. *J Am Acad Dermatol* 2019;81(1):297-305.
24. Hughes KL, Kirkham JJ, Clarke M, et al. Assessing the impact of a research funder's recommendation to consider core outcome sets. *PLoS One* 2019;14(9):e0222418. doi: 10.1371/journal.pone.0222418
25. OMERACT Initiative [Available from: https://omeract.org/ accessed cited 23 April 2019.
26. SONG Initiative [Available from: https://songinitiative.org/ accessed cited 23 April 2019.
27. CROWN Initiative [Available from: http://www.crown-initiative.org/ accessed cited 23 April 2019.
28. Borah R, Brown AW, Capers PL, et al. Analysis of the time and workers needed to conduct systematic reviews of medical interventions using data from the PROSPERO registry. *BMJ Open* 2017;7(2):e012545. doi: 10.1136/bmjopen-2016-012545
29. Tricco AC, Brehaut J, Chen MH, et al. Following 411 Cochrane protocols to completion: a retrospective cohort study. *PLoS One* 2008;3(11):e3684. doi: 10.1371/journal.pone.0003684

For peer review only

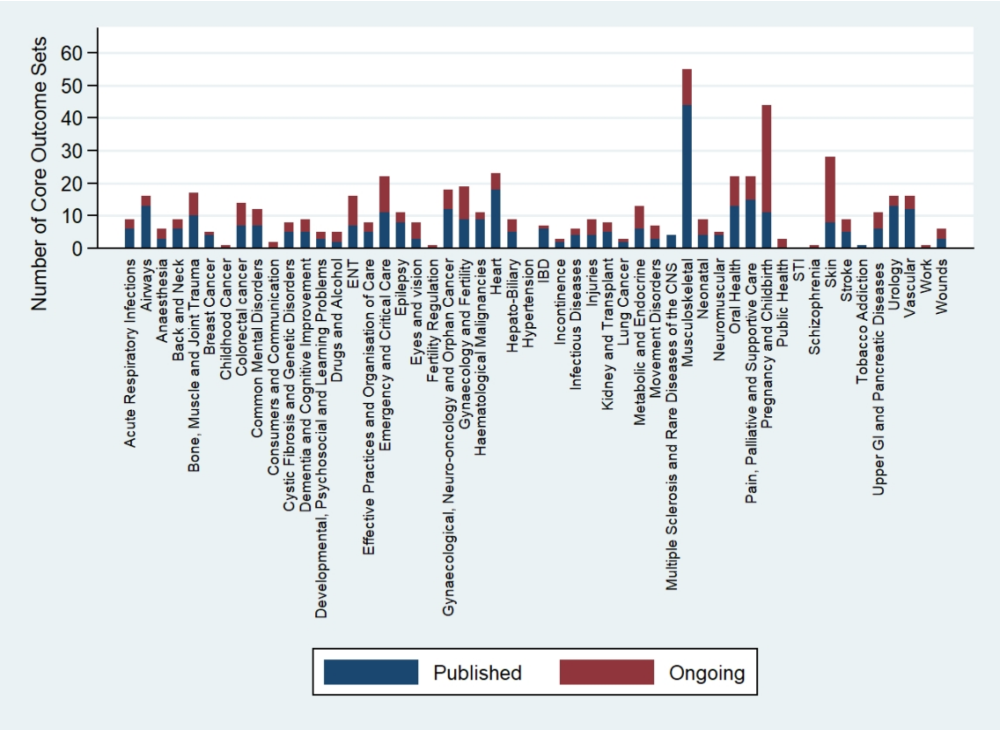

Figure 1. Published and Ongoing Core Outcome Sets according to Cochrane Review Group

108x78mm (300 x 300 DPI)

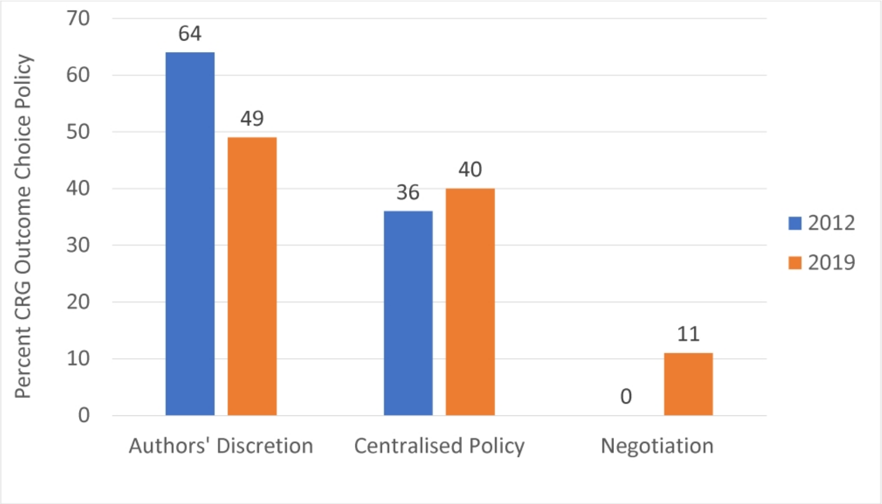

Figure 2. Cochrane Review Group Outcome Choice Policies. Denominators are (n=45) and (n=35) for 2012 and 2019 respectively.

297x194mm (300 x 300 DPI)

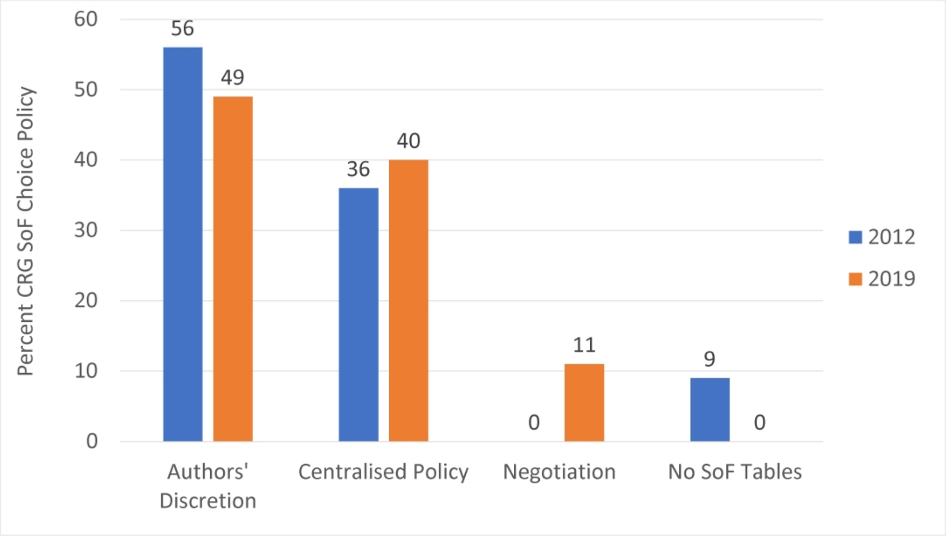

Figure 3. Cochrane Review Group SoF Table Choice Policies. Denominators are (n=45) and (n=35) for 2012 and 2019 respectively.

297x193mm (300 x 300 DPI)
